# Fuzzy Set Theory Applied on Autometrized Algebra

**DOI:** 10.12688/f1000research.161249.1

**Published:** 2025-02-04

**Authors:** Gebrie Yeshiwas Tilahun

**Affiliations:** 1 Department of Mathematics, Assosa University, Asosa, Benishangul-Gumuz, Ethiopia

**Keywords:** autometrized algebra, fuzzy subalgebra, fuzzy ideal, fuzzy congruence

## Abstract

This paper introduces fuzzy subalgebras of autometrized algebras and studies their properties. Also, we present fuzzy ideals of autometrized algebras and provide examples to illustrate our findings. We examine the homomorphisms of both the images and the inverse images of fuzzy subalgebras and ideals. Furthermore, we introduce fuzzy congruences on autometrized algebras. We prove that in normal autometrized algebra, the set of all fuzzy ideals is in one-to-one correspondence with the set of all fuzzy congruences.

## 1. Introduction

Ref. 1 introduced the notion of an autometrized algebra to obtain a unified theory of the then-known autometrized algebras: Boolean algebras, ^2^^,^^3^ Brouwerian algebras, ^4^ Newman algebras, ^5^ autometrized lattices ^4^ and commutative lattice ordered groups or l-groups. ^6^ Ideals and congruence in autometrized algebras were introduced and studied by Ref. 7. Further development of the autometrized algebra theory was done by Refs. 7– 14. Moreover, the notion of representable autometrized algebras explored by Refs. 15– 18.

In their research Refs. 19– 22, established the theory of subalgebras, ideals, and homomorphisms. They analyzed the link between normal autometrized l-algebras and representable autometrized algebras. Additionally Ref. 23, introduced the homomorphism, isomorphism, and correspondence theorems of autometrized algebra by using congruency, ^24^ introduced fuzzy sets, ^25^ presented a fuzzy group, ^26^ investigated fuzzy groups and fuzzy relations.

The previous studies did not investigate fuzzy subalgebra, fuzzy ideals, and fuzzy congruence of autometrized algebra. This paper introduces the concept of fuzzy subalgebras and fuzzy ideals of autometrized algebras. It will also present and prove various theorems and facts related to fuzzy subalgebras and fuzzy ideals of autometrized algebras. Additionally, the study includes fuzzy congruence relations on autometrized algebras.

The paper will have the following structure. Section 2 will provide definitions and terms. Section 3 will introduce the concept of fuzzy subalgebras of autometrized algebras. In Section 4, we introduce fuzzy ideals of autometrized algebras. Section 5 will cover fuzzy congruences of autometrized algebras and present many basic facts related to the idea. Finally, in Section 6, we will conclude the paper.

In this paper, A and B denote autometrized algebras (A,+,0,≤,⊗) and (B,+,0,≤,⊗) , respectively.

## 2. Preliminaries

In this section, we’ll look at some essential concepts, definitions, and terms; that are important in other sections.

Definition 2.1 ^1^ *A system* A=(A,+,0,≤,⊗) *is called an autometrized algebra if*

(i)
(A,+,0) *is a commutative monoid.*
(ii)
(A,≤) *is a partial ordered set, and* ≤ *is translation invariant, that is,* $\forall \gamma_{1},\;\gamma_{2},\;\gamma_{3}\in A;\;\gamma_{1}\leq \gamma_{2}\Rightarrow \gamma_{1}+\gamma_{3}\leq \gamma_{2}+\gamma_{3}$ .
(iii)
⊗:A×A→A *is autometric on* A *, that is,* ⊗ *satisfies metric operation axioms:*
  (a)
$\forall \gamma_{1},\;\gamma_{2}\in A;\;\gamma_{1}⊗\gamma_{2}\geq 0$ *and*, $\gamma_{1}⊗\gamma_{2}=0\Leftrightarrow \gamma_{1}=\gamma_{2}$ ,
  (b)
$\forall \gamma_{1},\;\gamma_{2}\in A;\;\gamma_{1}⊗\gamma_{2}=\gamma_{2}⊗\gamma_{1}$ ,
  (c)
$\forall \gamma_{1},\;\gamma_{2},\;\gamma_{3}\in A$ ; $\gamma_{1}⊗\gamma_{3}\leq \gamma_{1}⊗\gamma_{2}+\gamma_{2}⊗\gamma_{3}$ .

Definition 2.2 ^7^ A *is called normal if and only if*

(i)
$\gamma_{1}\leq \gamma_{1}⊗0\;\forall \gamma_{1}\in A$ .
(ii)
$\left(\gamma_{1}+\gamma_{3}\right)⊗\left(\gamma_{2}+\gamma_{4}\right)\leq \left(\gamma_{1}⊗\gamma_{2}\right)+\left(\gamma_{3}⊗\gamma_{4}\right)\;\forall \gamma_{1},\;\gamma_{2},\;\gamma_{3},\;\gamma_{4}\in A$ .
(iii)
$\left(\gamma_{1}⊗\gamma_{3}\right)⊗\left(\gamma_{2}⊗\gamma_{4}\right)\leq \left(\gamma_{1}⊗\gamma_{2}\right)+\left(\gamma_{3}⊗\gamma_{4}\right)\;\forall \gamma_{1},\;\gamma_{2},\;\gamma_{3},\;\gamma_{4}\in A$ .
(iv) *For any* $\gamma_{1}$ *and* $\gamma_{2}$ *in* A *,* $\gamma_{1}\leq \gamma_{2}\Rightarrow \exists \gamma_{3}\geq 0$ *such that* $\gamma_{1}+\gamma_{3}=\gamma_{2}$ .

Definition 2.3 ^19^ *Let* B⊆A *. Then* B *is said to be a subalgebra of* A *if;*

(i)
(B,+,0) *is a commutative monoid.*
(ii)
(B,≤) *is a subposet, and* ≤ *is translation invariant, that is,* $\gamma_{1}\leq \gamma_{2}\Rightarrow \gamma_{1}+\gamma_{3}\leq \gamma_{2}+\gamma_{3}$ *for any* $\gamma_{1},\;\gamma_{2},\;\gamma_{3}\in B$ .
(iii)
⊗|B:B×B→B *is metric.*

Definition 2.4 ^19^ *A nonempty subset* I *of* A *is called an ideal if and only if*

(i)
$\gamma_{1},\;\gamma_{2}\in I$ *imply* $\gamma_{1}+\gamma_{2}\in I$ .
(ii)
$\gamma_{1}\in I,\;\gamma_{2}\in A\;\text{and}\;\gamma_{2}⊗0\leq \gamma_{1}⊗0$ *imply* $\gamma_{2}\in I$ .

Definition 2.5 ^19^ *Every ideal of a normal autometrized algebra* A *is a subalgebra of* A .

Lemma 2.6 ^7^ *In a normal autometrized algebra, the intersection of any nonempty collection of ideals in* A *is again ideal.*

Definition 2.7 ^7^ A *is said to be semiregular if for any* γ∈A *,* γ≥0⇒γ⊗0=γ .

Definition 2.8 ^7^ *Let* A *is a normal autometrized algebra. An equivalence relation* Φ *on* A *is called a congruence relation if and only if*

(i)
$\left(\gamma_{1},\;\gamma_{2}\right),\;\left(\gamma_{3},\;\gamma_{4}\right)\in \Phi \Rightarrow \left(\gamma_{1}+\gamma_{3},\;\gamma_{2}+\gamma_{4}\right)\in \Phi \;\forall \gamma_{1},\;\gamma_{2},\;\gamma_{3},\;\gamma_{4}\in A$ ,
(ii)
$\left(\gamma_{1},\;\gamma_{2}\right),\;\left(\gamma_{3},\;\gamma_{4}\right)\in \Phi \Rightarrow \left(\gamma_{1}⊗\gamma_{3},\;\gamma_{2}⊗\gamma_{4}\right)\in \Phi \;\forall \gamma_{1},\;\gamma_{2},\;\gamma_{3},\;\gamma_{4}\in A$ ,
(iii)
$\left(\gamma_{3},\;\gamma_{4}\right)\in \Phi \;\text{and}\;\gamma_{1}⊗\gamma_{2}\leq \gamma_{3}⊗\gamma_{4}\Rightarrow \left(\gamma_{1},\;\gamma_{2}\right)\in \Phi \;\forall \gamma_{1},\;\gamma_{2},\;\gamma_{3},\;\gamma_{4}\in A$ .

Definition 2.9 ^19^ *Let* f:A→B *be a map. Then* f *is said to be a homomorphism from* A *to* B *if and only if*

(i) ( *i*) $f\left(\gamma_{1}+\gamma_{2}\right)=f\left(\gamma_{1}\right)+f\left(\gamma_{2}\right)\forall x,\;y\in A$ ,
(ii)
$f\left(\gamma_{1}⊗\gamma_{1}\right)=f\left(\gamma_{1}\right)⊗f\left(\gamma_{2}\right)\forall \gamma_{1},\;\gamma_{2}\in A$ *and*
(iii)
$\gamma_{1}\leq \gamma_{2}\Rightarrow f\left(\gamma_{1}\right)\leq f\left(\gamma_{2}\right)\forall \gamma_{1},\;\gamma_{2}\in A$ .

A homomorphism f:A→B is called an epimorphism if and only if f is onto.

Theorem 2.10 ^19^ *Let* f:A→B *be a homomorphism. Let* I *be an ideal of* B *. Then,* $N=f^{-1}\left(I\right)=\left\{\gamma \in A|f\left(\gamma \right)\in I\right\}$ *is an ideal of* A .

Theorem 2.11 ^19^ *Let* f:A→B *be an epimorphism and order-reversing. Let* I *be an ideal of* A *. Then,* L=f(I)={f(γ)∈B|γ∈I} *is an ideal of* B .

Theorem 2.12 ^19^ *Let* f:A→B *be a homomorphism. Then*

(a) *if* H *is a subalgebra of* A⇒f(H) *is a subalgebra of* B .
(b) *if* H *is a subalgebra of* $B\Rightarrow f^{-1}\left(H\right)$ *is a subalgebra of* A .

Definition 2.13 ^24^ *Let* A *be a nonempty set, a fuzzy subset* ζ *of* A *is a mapping* ζ:A→[0,1] .

Definition 2.14 ^24^ *Let* A *be a nonempty set and* μ *be a fuzzy subset of* A *, for* π∈[0,1] *, the set* $\zeta_{\pi }=\left\{\gamma \in A|\zeta \left(\gamma \right)\geq \pi \right\}$ *is called a level subset of* ζ .

Definition 2.15 ^25^ *A fuzzy subset* ζ *of a set* A *has* sup *property if for any subset* T *of* A *, there exist* $\pi_{0}\in T$ *such that* $\zeta \left(\pi_{0}\right)=\sup\left\{\zeta \left(\pi \right)|\pi \in T\right\}$ .

Definition 2.16 ^25^ *Let* f:A→B *be a mapping nonempty sets* A *and* B *respectively. If* ζ *is a fuzzy subset of* A *, then the fuzzy subset* α *of* B *defined by:*

$$f\left(\zeta \right)\left(\gamma_{2}\right)=\left\{ \begin{array}{ll} \sup\left\{\zeta \left(\gamma_{1}\right)|\gamma_{1}\in f^{-1}\left(\gamma_{2}\right)\right\}, & {\mathrm{iff}}^{-1}\left(\gamma_{2}\right)=\left\{\gamma_{1}\in A,f\left(\gamma_{1}\right)=\gamma_{2}\right\}\neq \emptyset .\\ 0, & \text{otherwise}. \end{array}\right.$$

*is said to be the image of* ζ *under* f .

*Similarly, if* α *is a fuzzy subset of* B *, then the fuzzy subset* ζ=(α◦f) *of* A *(that is, the fuzzy subset defined by* ζ(γ)=α(f(γ)) *for all* γ∈A *) is called the pre-image of* α *under* f .

## 3. Fuzzy subalgebra of autometrized algebra

In this section, we will introduce the fuzzy subalgebras of autometrized algebras and examine their properties.

Definition 3.1 *A fuzzy subset* ζ *of* A *is called a fuzzy subalgebra of* A *if for all* $\gamma_{1},\gamma_{2}\in A$ *;*

(i)
$\zeta \left(0\right)\geq \zeta \left(\gamma_{1}\right)$ .
(ii)
$\zeta \left(\gamma_{1}+\gamma_{2}\right)\geq \min\left\{\zeta \left(\gamma_{1}\right),\zeta \left(\gamma_{2}\right)\right\}$ .
(iii)
$\zeta \left(\gamma_{1}⊗\gamma_{2}\right)\geq \min\left\{\zeta \left(\gamma_{1}\right),\zeta \left(\gamma_{2}\right)\right\}$ .

Theorem 3.2 *Let* ζ *be a fuzzy subset of* A *. Then* ζ *is a fuzzy subalgebra of* A *if and only if for any* π∈[0,1] *,* $\zeta_{\pi }$ *is a subalgebra of* A .

Proof.
Assume that ζ is a fuzzy subalgebra of A .

(i) Since ζ(0)≥ζ(γ) for all γ∈A ; ζ(0)≥ζ(γ)≥π for all $\gamma \in \zeta_{\pi }$ . Therefore, $0\in \zeta_{\pi }$ .
(ii) Let $\gamma_{1},\gamma_{2}\in A$ . Suppose that $\gamma_{1},\gamma_{2}\in \zeta_{\pi }$ . So, $\zeta \left(\gamma_{1}\right)\geq \pi$ and $\zeta \left(\gamma_{2}\right)\geq \pi$ . Since ζ is a fuzzy subalgebra of A ; $\zeta \left(\gamma_{1}+\gamma_{2}\right)\geq \min\left\{\zeta \left(\gamma_{1}\right),\zeta \left(\gamma_{2}\right)\right\}\geq \pi$ . This implies that $\gamma_{1}+\gamma_{2}\in \zeta_{\pi }$ .
(iii) Let $\gamma_{1},\gamma_{2}\in A$ . Suppose that $\gamma_{1},\gamma_{2}\in \zeta_{\pi }$ . So, $\zeta \left(\gamma_{1}\right)\geq \pi$ and $\zeta \left(\gamma_{2}\right)\geq \pi$ . Since ζ is a fuzzy subalgebra of A ; $\zeta \left(\gamma_{1}⊗\gamma_{2}\right)\geq \min\left\{\zeta \left(\gamma_{1}\right),\zeta \left(\gamma_{2}\right)\right\}\geq \pi$ . This implies that $\gamma_{1}⊗\gamma_{2}\in \zeta_{\pi }$ . Hence $\zeta_{\pi }$ is a subalgebra of A .

Conversely, assume that $\zeta_{\pi }$ is a subalgebra of A . To show that ζ is a fuzzy subalgebra of A .

(i) To show that ζ(0)≥ζ(γ) for all γ∈A . Assume that it is false. Therefore, there exists $\gamma^{\prime }\in A$ such that ζ(0)<ζ(γ) . Take $\pi^{\prime }=\frac{\zeta \left(\gamma^{\prime }\right)+\zeta \left(0\right)}{2}$ . Then, $\zeta \left(0\right)<\pi^{\prime }$ and $0\leq \pi^{\prime }\leq \zeta \left(\gamma^{\prime }\right)\leq 1$ . Therefore, $\gamma^{\prime }\in \zeta_{\pi^{\prime }}$ . So, $\zeta_{\pi^{\prime }}\neq \emptyset$ . As $\zeta_{\pi^{\prime }}$ is a subalgebra of A , we have $0\in \zeta_{\pi^{\prime }}$ . So, $\zeta \left(0\right)\geq \pi^{\prime }$ . This is contradiction.
(ii) Let $\gamma_{1},\gamma_{2}\in A$ . Take $\pi =\min\left\{\zeta \left(\gamma_{1}\right),\zeta \left(\gamma_{1}\right)\right\}$ . Therefore, $\gamma_{1}\in \zeta_{\pi }$ and $\gamma_{2}\in \zeta_{\pi }$ . Since $\zeta_{\pi }$ is a subalgebra of A ; $\gamma_{1}+\gamma_{2}\in \zeta_{\pi }$ . As a result, $\zeta \left(\gamma_{1}+\gamma_{2}\right)\geq \pi =\min\zeta \left(\gamma_{1}\right),\zeta \left(\gamma_{2}\right)$ .
(iii) Let $\gamma_{1},\gamma_{2}\in A$ . Take $\pi =\min\left\{\zeta \left(\gamma_{1}\right),\zeta \left(\gamma_{2}\right)\right\}$ . Therefore, $\gamma_{1}\in \zeta_{\pi }$ and $\gamma_{2}\in \zeta_{\pi }$ . Since $\zeta_{\pi }$ is a subalgebra of A ; $\gamma_{1}⊗\gamma_{2}\in \zeta_{\pi }$ . As a result, $\zeta \left(\gamma_{1}⊗\gamma_{2}\right)\geq \pi =\min\left\{\zeta \left(\gamma_{1}\right),\zeta \left(\gamma_{2}\right)\right\}$ .

Hence ζ is a fuzzy subalgebra of A .

Theorem 3.3 *The intersection of any set of fuzzy subalgebras of* A *is also fuzzy subalgebra of* A .

Proof.
Let $\left\{\zeta_{i}|i\in I\right\}$ be a family of fuzzy subalgebras of autometrized algebra A . To show that $\cap_{i\in I}\zeta_{i}$ is an ideal. Then for any $\gamma_{1},\gamma_{2}\in A$ , i∈I ,

(a)
$$\begin{array}{l} \left(\cap_{i\in I}\zeta_{i}\right)\left(0\right)=\inf\left(\zeta_{i}\left(0\right)\right).\\ \;\geq \inf\left(\zeta_{i}\left(\gamma_{1}\right)\right).\\ \;=\left(\cap_{i\in I}\zeta_{i}\right)\left(\gamma_{1}\right). \end{array}$$
(b)
$$\begin{array}{l} \left(\cap_{i\in I}\zeta_{i}\right)\left(\gamma_{1}+\gamma_{2}\right)=\inf\left(\zeta_{i}\left(\gamma_{1}+\gamma_{2}\right)\right).\\ \;\geq \inf\left(\min\left\{\zeta_{i}\left(\gamma_{1}\right),\zeta_{i}\left(\gamma_{2}\right)\right\}\right).\\ \;=\min\left\{\inf\left(\zeta_{i}\left(\gamma_{1}\right)\right),\inf\left(\zeta_{i}\left(\gamma_{2}\right)\right)\right\}.\\ \;=\min\left\{\left(\cap_{i\in I}\zeta_{i}\right)\left(\gamma_{1}\right),\left(\cap_{i\in I}\zeta_{i}\right)\left(\gamma_{2}\right)\right\}. \end{array}$$
(c)
$$\begin{array}{l} \left(\cap_{i\in I}\zeta_{i}\right)\left(\gamma_{1}⊗\gamma_{2}\right)=\inf\left(\zeta_{i}\left(\gamma_{1}⊗\gamma_{2}\right)\right).\\ \;\geq \inf\left(\min\left\{\zeta_{i}\left(\gamma_{1}\right),\zeta_{i}\left(\gamma_{2}\right)\right\}\right).\\ \;=\min\left\{\inf\left(\zeta_{i}\left(\gamma_{1}\right)\right),\inf\left(\zeta_{i}\left(\gamma_{2}\right)\right)\right\}.\\ \;=\min\left\{\left(\cap_{i\in I}\zeta_{i}\right)\left(\gamma_{1}\right),\left(\cap_{i\in I}\zeta_{i}\right)\left(\gamma_{2}\right)\right\}. \end{array}$$

Remark 3.4 *The union of fuzzy subalgebras of* A *is not a fuzzy subalgebra of* A .

Example 3.5 *Let* $A=\left\{0,\;\gamma_{1},\;\gamma_{2},\;\gamma_{3}\right\}$ *with* $0\leq \gamma_{1},\;\gamma_{2}\leq \gamma_{3}$ *and elements* $\gamma_{1},\;\gamma_{2}$ *are incomparable. Define* ⊗ *and* + *by the following tables.*

**Table:** 

⊗	0	$\gamma_{1}$	$\gamma_{2}$	$\gamma_{3}$
0	0	$\gamma_{1}$	$\gamma_{2}$	$\gamma_{3}$
$\gamma_{1}$	$\gamma_{1}$	0	$\gamma_{3}$	$\gamma_{2}$
$\gamma_{2}$	$\gamma_{2}$	$\gamma_{3}$	0	$\gamma_{1}$
$\gamma_{3}$	$\gamma_{3}$	$\gamma_{2}$	$\gamma_{1}$	0

**Table:** 

+	0	$\gamma_{1}$	$\gamma_{2}$	$\gamma_{3}$
0	0	$\gamma_{1}$	$\gamma_{2}$	$\gamma_{3}$
$\gamma_{1}$	$\gamma_{1}$	$\gamma_{1}$	$\gamma_{3}$	$\gamma_{3}$
$\gamma_{2}$	$\gamma_{2}$	$\gamma_{3}$	$\gamma_{2}$	$\gamma_{3}$
$\gamma_{3}$	$\gamma_{3}$	$\gamma_{3}$	$\gamma_{3}$	$\gamma_{3}$

*Then,* A *is an autometrized algebra. Define a fuzzy subset* ζ *and* α *of* A *by:*

**Table:** 

	0	$\gamma_{1}$	$\gamma_{2}$	$\gamma_{3}$
ζ	0.5	0.3	0.5	0.3
α	0.5	0.5	0.4	0.4
ζ∪α	0.5	0.5	0.5	0.4

*Then* ζ *and* α *are fuzzy subalgebras of* A *. But the union* ζ∪α *is not a fuzzy subalgebras of* A *. Since* $\zeta \cup \alpha \left(\gamma_{1}⊗\gamma_{2}\right)=\zeta \cup \alpha \left(\gamma_{3}\right)=0.4<0.5=\min\left\{\zeta \cup \alpha \left(\gamma_{1}\right),\zeta \cup \alpha \left(\gamma_{2}\right)\right\}$ .

Remark 3.6 *The union of any chain set of fuzzy subalgebras of* A *is also fuzzy subalgebra of* A .

Theorem 3.7 *A homomorphic pre-image of a fuzzy subalgebra of* A *is also a fuzzy subalgebra of* A .

Proof.
Let f:A→B be a homomorphism. Let α a fuzzy subalgebra of B and ζ the pre-image of α under f , then α(f(γ))=ζ(γ) for all γ∈A . To show that ζ is a fuzzy subalgebra of A .

(i) Since f(γ)∈B and α is a fuzzy subalgebra of B ; $\alpha \left(0^{\prime }\right)\geq \alpha \left(f\left(\gamma \right)\right)=\zeta \left(\gamma \right)$ , for every γ∈A , where $0^{\prime }$ is the zero element of B . But $\alpha \left(0^{\prime }\right)=\alpha \left(f\left(0\right)\right)=\zeta \left(0\right)$ . Therefore, ζ(0)≥ζ(γ) for any γ∈A .
(ii) Let $\gamma_{1},\gamma_{2}\in A$ .

$$\begin{array}{l} \zeta \left(\gamma_{1}+\gamma_{2}\right)=\alpha \left(f\left(\gamma_{1}+\gamma_{2}\right)\right).\\ \;=\alpha \left(f\left(\gamma_{1}\right)+f\left(\gamma_{2}\right)\right).\\ \;\geq \min\left\{\alpha \left(f\left(\gamma_{1}\right)\right),\alpha \left(f\left(\gamma_{2}\right)\right)\right\}.\\ \;=\min\left\{\zeta \left(\gamma_{1}\right),\zeta \left(\gamma_{2}\right)\right\}. \end{array}$$

(iii) Let $\gamma_{1},\gamma_{2}\in A$ .

$$\begin{array}{l} \zeta \left(\gamma_{1}⊗\gamma_{2}\right)=\alpha \left(f\left(\gamma_{1}⊗\gamma_{2}\right)\right).\\ \;=\alpha \left(f\left(\gamma_{1}\right)⊗f\left(\gamma_{2}\right)\right).\\ \;\geq \min\left\{\alpha \left(f\left(\gamma_{1}\right)\right),\alpha \left(f\left(\gamma_{2}\right)\right)\right\}.\\ \;=\min\left\{\zeta \left(\gamma_{1}\right),\zeta \left(\gamma_{2}\right)\right\}. \end{array}$$

Hence, ζ is a fuzzy subalgebra of A .

Theorem 3.8 *Let* f:A→B *be an epimorphism. For every fuzzy subalgebra* ζ *of* A *with sup property,* f(ζ) *is a fuzzy subalgebra of* B .

Proof.
To show that f(ζ) is a fuzzy subalgebra of B . By definition $\alpha \left(\gamma_{2}^{\prime }\right)=f\left(\zeta \right)\left(\gamma_{2}^{\prime }\right)=\sup\left\{\zeta \left(\gamma_{1}\right)|\gamma_{1}\in f^{-1}\left(\gamma_{2}^{\prime }\right)\right\}$ , for all $\gamma_{2}^{\prime }\in B$ ( sup∅=0 ).

(i) Since ζ is a fuzzy subalgebra of A ; we have ζ(0)≥ζ(γ) , for every γ∈A . We know that $0\in f^{-1}\left(0^{\prime }\right)$ , where 0 and $0^{\prime }$ are the zero elements of A and B respectively. Therefore, $\alpha \left(0^{\prime }\right)=\sup_{\pi \in f^{-1}\left(0^{\prime }\right)}\zeta \left(\pi \right)=\zeta \left(0\right)\geq \zeta \left(\gamma \right)$ for all γ∈A . Which implies that: $\alpha \left(0^{\prime }\right)\geq \sup_{\pi \in f^{-1}\left(\gamma^{\prime }\right)}\zeta \left(\pi \right)=\alpha \left(\gamma^{\prime }\right)$ for any $\gamma^{\prime }\in B$ .
(ii) Let $\gamma^{\prime },\eta^{\prime }\in B$ . Then, there exist $\gamma_{0},\eta_{0}\in A$ such that $f\left(\gamma_{0}\right)=\gamma^{\prime }$ and $f\left(\eta_{0}\right)=\eta^{\prime }$ . So, $\gamma_{0}\in f^{-1}\left(\gamma^{\prime }\right)$ and $\eta_{0}\in f^{-1}\left(\eta^{\prime }\right)$ . Now, consider
$$\begin{array}{l} \zeta \left(\gamma_{0}+\eta_{0}\right)=\alpha \left(f\left(\gamma_{0}+\eta_{0}\right)\right)=\alpha \left(f\left(\gamma_{0}\right)+f\left(\eta_{0}\right)\right)=\alpha \left(\gamma^{\prime }+\eta^{\prime }\right)=\underset{\gamma_{0}+\eta_{0}\in f^{-1}\left(\gamma^{\prime }+\eta^{\prime }\right)}{\sup}\zeta \left(\gamma_{0}+\eta_{0}\right).\\ \zeta \left(\gamma_{0}\right)=\alpha \left(f\left(\gamma_{0}\right)\right)=\alpha \left(\gamma^{\prime }\right)=\underset{\gamma_{0}\in f^{-1}\left(\gamma^{\prime }\right)}{\sup}\zeta \left(\gamma_{0}\right). \end{array}$$
  Also,
$$\begin{array}{l} \alpha \left(\gamma^{\prime }⊗\eta^{\prime }\right)=\underset{\pi \in f^{-1}\left(\gamma^{\prime }+\eta^{\prime }\right)}{\sup}\zeta \left(\pi \right).\\ \geq \min\left\{\zeta \left(\gamma_{0}\right),\zeta \left(\eta_{0}\right)\right\}.\\ =\min\left\{\underset{\pi \in f^{-1}\left(\gamma^{\prime }\right)}{\sup}\zeta \left(\gamma_{0}\right),\underset{\pi \in f^{-1}\left(\eta^{\prime }\right)}{\sup}\zeta \left(\eta_{0}\right)\right\}.\\ =\min\left\{\alpha \left(\gamma^{\prime }\right),\alpha \left(\eta^{\prime }\right)\right\}. \end{array}$$
(iii) Let $\gamma^{\prime },\eta^{\prime }\in B$ . Then, there exist $\gamma_{0},\eta_{0}\in A$ such that $f\left(\gamma_{0}\right)=\gamma^{\prime }$ and $f\left(\eta_{0}\right)=\eta^{\prime }$ . So, $\gamma_{0}\in f^{-1}\left(\gamma^{\prime }\right)$ and $\eta_{0}\in f^{-1}\left(\eta^{\prime }\right)$ . Now, consider
$$\begin{array}{l} \zeta \left(\gamma_{0}⊗\eta_{0}\right)=\alpha \left(f\left(\gamma_{0}⊗\eta_{0}\right)\right)=\alpha \left(f\left(\gamma_{0}\right)⊗f\left(\eta_{0}\right)\right)=\alpha \left(\gamma^{\prime }⊗\eta^{\prime }\right)=\underset{\gamma_{0}⊗\eta_{0}\in f^{-1}\left(\gamma^{\prime }⊗\eta^{\prime }\right)}{\sup}\zeta \left(\gamma_{0}⊗\eta_{0}\right).\\ \zeta \left(\gamma_{0}\right)=\alpha \left(f\left(\gamma_{0}\right)\right)=\alpha \left(\gamma^{\prime }\right)=\underset{\gamma_{0}\in f^{-1}\left(\gamma^{\prime }\right)}{\sup}\zeta \left(\gamma_{0}\right). \end{array}$$
  Also,
$$\begin{array}{l} \alpha \left(\gamma^{\prime }⊗\eta^{\prime }\right)=\underset{\pi \in f^{-1}\left(\gamma^{\prime }⊗\eta^{\prime }\right)}{\sup}\zeta \left(\pi \right).\\ \;\geq \min\left\{\zeta \left(\gamma_{0}\right),\zeta \left(\eta_{0}\right)\right\}.\\ \;=\min\left\{\underset{\pi \in f^{-1}\left(\gamma^{\prime }\right)}{\sup}\zeta \left(\gamma_{0}\right),\underset{\pi \in f^{-1}\left(\eta^{\prime }\right)}{\sup}\zeta \left(\eta_{0}\right)\right\}.\\ \;=\min\left\{\alpha \left(\gamma^{\prime }\right),\alpha \left(\eta^{\prime }\right)\right\}. \end{array}$$

Hence, α is a fuzzy subalgebra of B .

## 4. Fuzzy ideals of autometrized algebra

This section introduces the notion of fuzzy ideals of autometrized algebras and examines several basic properties related to fuzzy ideals.

Definition 4.1 *A fuzzy subset* ζ *of* A *is called a fuzzy ideal of* A *if it satisfies the following conditions: for all* $\gamma_{1},\gamma_{2}\in A$ *;*

(i)
$\zeta \left(\gamma_{1}+\gamma_{2}\right)\geq \min\left\{\zeta \left(\gamma_{1}\right),\zeta \left(\gamma_{2}\right)\right\}$ .
(ii) *if* $\gamma_{1}⊗0\leq \gamma_{2}⊗0$ *, then* $\zeta \left(\gamma_{1}\right)\geq \zeta \left(\gamma_{2}\right)$ .

Example 4.2 *Let* $A=\left\{0,\;\gamma_{1},\;\gamma_{2},\;\gamma_{3}\right\}$ *with* $0\leq \gamma_{1},\;\gamma_{2}\leq \gamma_{3}$ *and elements* $\gamma_{1},\;\gamma_{2}$ *are incomparable. Define* ⊗ *and* + *by the following tables.*

**Table:** 

⊗	0	$\gamma_{1}$	$\gamma_{2}$	$\gamma_{3}$
0	0	$\gamma_{1}$	$\gamma_{2}$	$\gamma_{3}$
$\gamma_{1}$	$\gamma_{1}$	0	$\gamma_{3}$	$\gamma_{2}$
$\gamma_{2}$	$\gamma_{2}$	$\gamma_{3}$	0	$\gamma_{1}$
$\gamma_{3}$	$\gamma_{3}$	$\gamma_{2}$	$\gamma_{1}$	0

**Table:** 

+	0	$\gamma_{1}$	$\gamma_{2}$	$\gamma_{3}$
0	0	$\gamma_{1}$	$\gamma_{2}$	$\gamma_{3}$
$\gamma_{1}$	$\gamma_{1}$	$\gamma_{1}$	$\gamma_{3}$	$\gamma_{3}$
$\gamma_{2}$	$\gamma_{2}$	$\gamma_{3}$	$\gamma_{2}$	$\gamma_{3}$
$\gamma_{3}$	$\gamma_{3}$	$\gamma_{3}$	$\gamma_{3}$	$\gamma_{3}$

*Then,* A *is an autometrized algebra. Define a fuzzy subset* ζ:A→[0,1] *by,* $\zeta \left(0\right)=\zeta \left(\gamma_{1}\right)=0.5$ *and* $\zeta \left(\gamma_{2}\right)=\zeta \left(\gamma_{3}\right)=0.2$ *. Here,* $ℐ=\left\{0,\;\gamma_{1}\right\}$ *is an ideal of* A *. It is clear that* ζ *is a fuzzy ideal of* A .

Theorem 4.3 *Let* ζ *be a fuzzy subset of* A *. Then* ζ *is a fuzzy ideal of* A *if and only if for any* π∈[0,1] *,* $\zeta_{\pi }$ *is an ideal of* A .

Proof.
Assume that ζ is a fuzzy ideal of A .

(i) Since 0≤γ⊗0 for any γ∈A ; by properties of fuzzy ideal ζ(0)≥ζ(γ) for all γ∈A . Therefore, ζ(0)≥ζ(γ)≥π for $\gamma \in \zeta_{\pi }$ . Therefore, $0\in \zeta_{\pi }$ .
(ii) Let $\gamma_{1},\gamma_{2}\in A$ . Suppose that $\gamma_{1},\gamma_{2}\in \zeta_{\pi }$ . So, $\zeta \left(\gamma_{1}\right)\geq \pi$ and $\zeta \left(\gamma_{2}\right)\geq \pi$ . Since ζ is a fuzzy ideal of A ; $\zeta \left(\gamma_{1}+\gamma_{2}\right)\geq \min\left\{\zeta \left(\gamma_{1}\right),\zeta \left(\gamma_{2}\right)\right\}\geq \pi$ . This implies that $\gamma_{1}+\gamma_{2}\in \zeta_{\pi }$ .
(iii) Let $\gamma_{1},\gamma_{2}\in A$ such that $\gamma_{1}\in \zeta_{\pi }$ . suppose $\gamma_{2}⊗0\leq \gamma_{1}⊗0$ . Clearly, $\zeta \left(\gamma_{1}\right)\geq \pi$ . Since ζ is a fuzzy ideal of A ; $\zeta \left(\gamma_{2}\right)\geq \zeta \left(\gamma_{1}\right)\geq \pi$ . This implies that $\gamma_{2}\in \zeta_{\pi }$ . Hence $\zeta_{\pi }$ is a an ideal of A .

Conversely, assume that $\zeta_{\pi }$ is an ideal of A . To show that ζ is a fuzzy ideal of A .

(i) Let $\gamma_{1},\gamma_{2}\in A$ . Take $\pi =\min\left\{\zeta \left(\gamma_{1}\right),\zeta \left(\gamma_{1}\right)\right\}$ . Therefore, $\gamma_{1}\in \zeta_{\pi }$ and $\gamma_{2}\in \zeta_{\pi }$ . Since $\zeta_{\pi }$ is an ideal of A ; $\gamma_{1}+\gamma_{2}\in \zeta_{\pi }$ . As a result, $\zeta \left(\gamma_{1}+\gamma_{2}\right)\geq \pi =\min\zeta \left(\gamma_{1}\right),\zeta \left(\gamma_{2}\right)$ .
(ii) Let $\gamma_{1},\gamma_{2}\in A$ . Suppose $\gamma_{1}⊗0\leq \gamma_{2}⊗0$ . Take $\pi =\zeta \left(\gamma_{2}\right)$ . So, $\gamma_{2}\in \zeta_{\pi }$ . Since $\zeta_{\pi }$ is an ideal; $\gamma_{1}\in \zeta_{\pi }$ . As a result, $\zeta \left(\gamma_{1}\right)\geq \pi =\zeta \left(\gamma_{2}\right)$ .

Hence ζ is a fuzzy ideal of A .

Theorem 4.4 *Let* ζ *be a fuzzy set of a normal autometrized algebra* A *. Define a fuzzy set* α *as:* $\alpha \left(x\right)=\sup\left\{\min\left\{\zeta \left(\gamma_{1}\right),\ldots ,\zeta \left(\gamma_{n}\right)\right\}|x⊗0\leq m_{1}\left(\gamma_{1}⊗0\right)+\ldots +m_{k}\left(\gamma_{k}⊗0\right)\text{for}\;\text{some}\;\text{positive}\;\text{integers}\;m_{1},\ldots ,m_{k}\;\text{and}\;\gamma_{1},\ldots ,\gamma_{k}\in A\right\}$ .

*Then* α *is the smallest fuzzy ideal of* A *that contains* ζ .

Proof.
Let x,y∈A . This implies

$$x⊗0\leq m_{1}\left(\gamma_{1}⊗0\right)+\ldots +m_{k}\left(\gamma_{k}⊗0\right)$$

and,

$$y⊗0\leq n_{1}\left(\eta_{1}⊗0\right)+\ldots +n_{l}\left(\eta_{l}⊗0\right)$$

for $m_{1},\ldots ,m_{k},\;n_{1},\ldots ,n_{l}$ are positive integers and $\gamma_{i},\;\eta_{i}\in A$ . By property of normal; we get:

$$\begin{array}{l} \left(x+y\right)⊗0\leq \left(x⊗0\right)+\left(y⊗0\right)\\ \leq m_{1}\left(\gamma_{1}⊗0\right)+\ldots +m_{k}\left(\gamma_{k}⊗0\right)+n_{1}\left(\eta_{1}⊗0\right)+\ldots +n_{l}\left(\eta_{l}⊗0\right) \end{array}$$

for $m_{1},\ldots ,m_{k},\;n_{1},\ldots ,n_{l}$ are positive integers and $\gamma_{i},\;\eta_{i}\in A$ .

Therefore $\alpha \left(x+y\right)\geq \min\left\{\zeta \left(\gamma_{1}\right),\ldots ,\zeta \left(\gamma_{n}\right),\zeta \left(\eta_{1}\right),\ldots ,\zeta \left(\eta_{l}\right)\right\}$ .

Denote by $H=\left\{\min\left\{\zeta \left(\eta_{1}\right),\ldots ,\zeta \left(\eta_{l}\right)\right\}|y⊗0\leq n_{1}\left(\eta_{1}⊗0\right)+\ldots +n_{l}\left(\eta_{l}⊗0\right)\;\text{for}\;\text{some}\;\text{positive}\;\text{integers}n_{1},\ldots ,n_{l}\;\text{and}\;\eta_{1},\ldots ,\eta_{l}\in A\right\}$ .

And $J=\{\min\left\{\zeta \left(\gamma_{1}\right),\ldots ,\zeta \left(\gamma_{n}\right)\right\}|x⊗0\leq m_{1}\left(\gamma_{1}⊗0\right)+\ldots +n_{k}\left(\gamma_{k}⊗0\right)\;\text{for}\;\text{some}\;\text{positive}\;\text{integers}\;m_{1},\ldots ,m_{k}$ $\text{and}\;\gamma_{1},\ldots ,\gamma_{k}\in A\}$ .

We have $\min\left\{\alpha \left(x\right),\alpha \left(y\right)\right\}=\min\left\{\sup H,\;\sup J\right\}=\sup\min\left\{\zeta \left(\eta_{1}\right),\ldots ,\zeta \left(\eta_{l}\right),\;\zeta \left(\gamma_{1}\right),\ldots ,\zeta \left(\gamma_{n}\right)\right\}|y⊗0\leq n_{1}\left(\eta_{1}⊗0\right)+\ldots +n_{l}\left(\eta_{l}⊗0\right),\;x⊗0\leq m_{1}\left(\gamma_{1}⊗0\right)+\ldots +n_{k}\left(\gamma_{k}⊗0\right).$ for some positive integers $m_{1},\ldots ,m_{k},n_{1},\ldots ,n_{l}\;\text{and}\;\eta_{1},\ldots ,\eta_{l},\gamma_{1},\ldots ,\gamma_{k}\in A$ . Hence, α(x+y)≥min{α(x),α(y)} .

Let x,y∈A and y⊗0≤x⊗0 . Also,

$$x⊗0\leq m_{1}\left(\gamma_{1}⊗0\right)+\ldots +m_{k}\left(\gamma_{k}⊗0\right)$$

and,

$$y⊗0\leq n_{1}\left(\eta_{1}⊗0\right)+\ldots +n_{l}\left(\eta_{l}⊗0\right)$$

for $m_{1},\ldots ,m_{k},\;n_{1},\ldots ,n_{l}$ are positive integers and $\gamma_{i},\;\eta_{i}\in A$ . Clearly,

$$y⊗0\leq m_{1}\left(\gamma_{1}⊗0\right)+\ldots +m_{k}\left(\gamma_{k}⊗0\right)+n_{1}\left(\eta_{1}⊗0\right)+\ldots +n_{l}\left(\eta_{l}⊗0\right).$$

Therefore, $\alpha \left(y\right)=\sup\{\min\left\{\zeta \left(\eta_{1}\right),\ldots ,\zeta \left(\eta_{l}\right),\;\zeta \left(\gamma_{1}\right),\ldots ,\zeta \left(\gamma_{n}\right)\right\}|y⊗0\leq m_{1}\left(\gamma_{1}⊗0\right)+\ldots +m_{k}\left(\gamma_{k}⊗0\right)+n_{1}\left(\eta_{1}⊗0\right)+\ldots +n_{l}\left(\eta_{l}⊗0\right)\;$ for some positive integers $\;m_{1},\ldots ,m_{k},n_{1},\ldots ,n_{l}\;\text{and}\eta_{1},\ldots ,\eta_{l},\gamma_{1},\ldots ,\gamma_{k}\in A\}$ . And hence α(y)≥α(x) . Therefore, α is a fuzzy ideal.

Let γ∈A . We know that γ⊗0≤1(γ⊗0) . We have α(γ)≥min{ζ(γ),ζ(γ)}=ζ(γ) . That is α contains ζ .

Let μ be a fuzzy ideal of A containing ζ . To show that α(x)≤μ(x) . Let x∈A . Therefore,

$\alpha \left(x\right)=\sup\min\left\{\zeta \left(\gamma_{1}\right),\ldots ,\zeta \left(\gamma_{n}\right)\right\}|x⊗0\leq m_{1}\left(\gamma_{1}⊗0\right)+\ldots +m_{k}\left(\gamma_{k}⊗0\right)\;$ for some positive integers $m_{1},\ldots ,m_{k}\;\text{and}\;\gamma_{1},\ldots ,\gamma_{k}\in A\leq \sup\min\left\{\mu \left(\gamma_{1}\right),\ldots ,\mu \left(\gamma_{n}\right)\right\}|x⊗0\leq m_{1}\left(\gamma_{1}⊗0\right)+\ldots +m_{k}\left(\gamma_{k}⊗0\right)$ for some positive integers $m_{1},\ldots ,m_{k}\;\text{and}\;\gamma_{1},\ldots ,\gamma_{k}\in A\leq \mu \left(x\right)$ . Hence α is the smallest fuzzy ideal of A that containing ζ .

Let I be a nonempty subset of A and α,β∈[0,1] such that α>β . Now we define fuzzy set $\zeta_{I}$ by

$$\zeta_{I}\left(a\right)=\left\{ \begin{array}{ll} \alpha , & \text{if}\;a\in I.\\ \beta , & \text{otherwise}. \end{array}\right.$$

Theorem 4.5 *Let* I *be a nonempty subset of* A *. Then* $\zeta_{ℐ}$ *is a fuzzy ideal of* A *if and only if* I *is an ideal of* A .

Proof.
Assume that $\zeta_{ℐ}$ is a fuzzy ideal of A .

( *i*) Let $\gamma_{1},\gamma_{2}\in A$ . If $\gamma_{1},\gamma_{2}\in I$ , then $\zeta_{ℐ}\left(\gamma_{1}\right)=\zeta_{ℐ}\left(\gamma_{2}\right)=\alpha$ . So, $\zeta_{ℐ}\left(\gamma_{1}+\gamma_{2}\right)\geq \min\left\{\zeta_{ℐ}\left(\gamma_{1}\right),\zeta_{ℐ}\left(\gamma_{2}\right)\right\}=\alpha$ . Therefore, $\gamma_{1}+\gamma_{2}\in I$ .

( *iii*) Let $\gamma_{1}\in I$ and $\gamma_{2}\in A$ . Suppose $\gamma_{2}⊗0\leq \gamma_{1}⊗0$ . Clearly, $\zeta_{ℐ}\left(\gamma_{1}\right)=\alpha$ . Since $\zeta_{ℐ}\left(\gamma_{2}\right)\geq \zeta_{ℐ}\left(\gamma_{1}\right)\geq \alpha$ . Clearly, $\zeta_{ℐ}\left(\gamma_{2}\right)=\alpha$ . Therefore, $\gamma_{2}\in I$ .

Conversely, let I is an ideal of A .

( *a*) Let $\gamma_{1},\gamma_{2}\in A$ . Consider the following three cases.

( *i*) If $\gamma_{1},\gamma_{2}\in I$ , then $\gamma_{1}+\gamma_{2}\in I$ . Clearly, $\zeta_{ℐ}\left(\gamma_{1}\right)=\zeta_{ℐ}\left(\gamma_{2}\right)=\alpha$ . So, $\zeta_{ℐ}\left(\gamma_{1}+\gamma_{2}\right)=\alpha =\min\left\{\zeta_{ℐ}\left(\gamma_{1}\right),\zeta_{ℐ}\left(\gamma_{2}\right)\right\}$ .

( *ii*) If $\gamma_{1}\in I$ or $\gamma_{2}\notin I$ . Then $\zeta_{ℐ}\left(\gamma_{1}\right)=\alpha$ and $\zeta_{ℐ}\left(\gamma_{2}\right)=\beta$ . Clearly, $\zeta_{ℐ}\left(\gamma_{1}+\gamma_{2}\right)\geq \beta =\min\left\{\zeta_{ℐ}\left(\gamma_{1}\right),\zeta_{ℐ}\left(\gamma_{2}\right)\right\}$ .

( *iii*) If $\gamma_{1}\in I$ or $\gamma_{2}\in I$ . Then $\zeta_{ℐ}\left(\gamma_{1}\right)=\beta$ or $\zeta_{ℐ}\left(\gamma_{2}\right)=\beta$ . Clearly, $\zeta_{ℐ}\left(\gamma_{1}+\gamma_{2}\right)\geq \beta =\min\left\{\zeta_{ℐ}\left(\gamma_{1}\right),\zeta_{ℐ}\left(\gamma_{2}\right)\right\}$ . Therefore, from Case - i, Case - ii and Case - iii, we get $\zeta_{ℐ}\left(\gamma_{1}+\gamma_{2}\right)\geq \beta =\min\left\{\zeta_{ℐ}\left(\gamma_{1}\right),\zeta_{ℐ}\left(\gamma_{2}\right)\right\}$ for any $\gamma_{1},\gamma_{2}\in A$ .

( *b*) Let $\gamma_{1},\gamma_{2}\in A$ and $\gamma_{1}⊗0\leq \gamma_{2}⊗0$ . Consider the following three cases.

( *i*) If $\gamma_{2}\in I$ , then $\gamma_{1}\in I$ . Therefore, $\zeta_{ℐ}\left(\gamma_{2}\right)=\zeta_{ℐ}\left(\gamma_{1}\right)=\alpha$ .

( *ii*) If $\gamma_{2}\notin I$ , then $\zeta_{ℐ}\left(\gamma_{2}\right)=\beta$ . Thus, $\zeta_{ℐ}\left(\gamma_{1}\right)\geq \zeta_{ℐ}\left(\gamma_{2}\right)=\beta$ .

( *iii*) If $\gamma_{1},\;\gamma_{2}\notin I$ , then $\zeta_{ℐ}\left(\gamma_{1}\right)=\zeta_{ℐ}\left(\gamma_{2}\right)=\beta$ . Thus, $\zeta_{ℐ}\left(\gamma_{1}\right)=\zeta_{ℐ}\left(\gamma_{2}\right)=\beta$ . Consequently, for any $\gamma_{1},\gamma_{2}\in A$ and $\gamma_{1}⊗0\leq \gamma_{2}⊗0$ , we get $\zeta_{ℐ}\left(\gamma_{1}\right)\geq \zeta_{ℐ}\left(\gamma_{2}\right)$ . So, $\zeta_{ℐ}$ is a fuzzy ideal.

Theorem 4.6 *Let* ζ *be a fuzzy ideal of* A *. Then the set* $I_{0}=\left\{\gamma \in A|\zeta \left(\gamma \right)=\zeta \left(0\right)\right\}$ *is an ideal of* A .

Proof.

(a) Let $\gamma_{1},\gamma_{2}\in I_{0}$ . So, $\zeta \left(\gamma_{1}\right)=\zeta \left(0\right)$ and $\zeta \left(\gamma_{2}\right)=\zeta \left(0\right)$ . Since ζ is a fuzzy ideal of A ; $\zeta \left(\gamma_{1}+\gamma_{2}\right)\geq \min\left\{\zeta \left(\gamma_{1}\right),\zeta \left(\gamma_{2}\right)\right\}\geq \zeta \left(0\right)$ . Also, since ζ(0)≥ζ(γ) for any γ∈A , we get $\zeta \left(0\right)\geq \zeta \left(\gamma_{1}+\gamma_{2}\right)$ . Consequently, $\zeta \left(\gamma_{1}+\gamma_{2}\right)=\zeta \left(0\right)$ . Therefore, $\gamma_{1}+\gamma_{2}\in I_{0}$ .
(b) Let $\gamma_{1}\in I_{0}$ and $\gamma_{2}\in A$ . Clearly, $\zeta \left(\gamma_{1}\right)=\zeta \left(0\right)$ . Suppose $\gamma_{2}⊗0\leq \gamma_{1}⊗0$ . Since ζ is a fuzzy ideal of A ; $\zeta \left(\gamma_{2}\right)\geq \zeta \left(\gamma_{1}\right)=\zeta \left(0\right)$ . Also, since ζ(0)≥ζ(γ) for any γ∈A , we get $\zeta \left(0\right)\geq \zeta \left(\gamma_{2}\right)$ . As a result, $\zeta \left(\gamma_{2}\right)=\zeta \left(0\right)$ . Therefore, $\gamma_{2}\in I_{0}$ .

Hence, $I_{0}$ is an ideal of A .

Theorem 4.7 *Let* ζ *be a fuzzy ideal of* A *. Let* $\zeta_{\alpha }$ *,* $\zeta_{\beta }$ *be level ideals of* ζ *. Assume that* β<α *. Then,* $\zeta_{\alpha }$ *=* $\zeta_{\beta }$ *if and only if there is no* γ∈A *such that* β≤ζ(γ)<α .

Proof.
Assume that $\zeta_{\alpha }$ = $\zeta_{\beta }$ . Suppose for β≤α , there exists γ∈A such that β≤ζ(γ)<α . Then $\zeta_{\alpha }$ is a proper subset of $\zeta_{\beta }$ . This is contradiction. Hence, there is no γ∈A such that β≤ζ(γ)<α .

Conversely, suppose there is no γ∈A such that β≤ζ(γ)<α . Clearly, β≤α . Therefore, $\zeta_{\alpha }\subseteq \zeta_{\beta }$ . Let $\eta \in \zeta_{\beta }$ . Then ζ(η)≥β . Since ζ(η) doesnot lie between β and α ; ζ(η)≥α . Therefore, $\eta \in \zeta_{\alpha }$ . This implies that $\zeta_{\beta }\subseteq \zeta_{\alpha }$ . Hence, $\zeta_{\alpha }$ = $\zeta_{\beta }$ .

Theorem 4.8 *The intersection of any set of fuzzy ideals of* A *is also fuzzy ideal of* A .

Proof.
Let $\left\{\zeta_{i}|i\in I\right\}$ be a family of fuzzy subalgebras of autometrized algebra A . To show that $\cap_{i\in I}\zeta_{i}$ is an ideal. Then for any $\gamma_{1},\;\gamma_{2}\in A$ , i∈I ,

(a)
$$\begin{array}{l} \left(\cap_{i\in I}\zeta_{i}\right)\left(\gamma_{1}+\gamma_{2}\right)=\inf\left(\zeta_{i}\left(\gamma_{1}+\gamma_{2}\right)\right).\\ \;\geq \inf\left(\min\left\{\zeta_{i}\left(\gamma_{1}\right),\zeta_{i}\left(\gamma_{2}\right)\right\}\right).\\ \;=\min\left\{\inf\left(\zeta_{i}\left(\gamma_{1}\right)\right),\inf\left(\zeta_{i}\left(\gamma_{2}\right)\right)\right\}.\\ \;=\min\left\{\left(\cap_{i\in I}\zeta_{i}\right)\left(\gamma_{1}\right),\left(\cap_{i\in I}\zeta_{i}\right)\left(\gamma_{2}\right)\right\}. \end{array}$$
(b) Let $\gamma_{1},\;\gamma_{2}\in A$ and $\gamma_{1}⊗0\leq \gamma_{2}⊗0$ .
$$\begin{array}{l} \Rightarrow \zeta_{i}\left(\gamma_{1}\right)\geq \zeta_{i}\left(\gamma_{2}\right).\\ \Rightarrow \inf\left(\zeta_{i}\left(\gamma_{1}\right)\right)\geq \inf\left(\zeta_{i}\left(\gamma_{2}\right)\right).\\ \Rightarrow \cap_{i\in I}\zeta_{i}\left(\gamma_{1}\right)\geq \cap_{i\in I}\zeta_{i}\left(\gamma_{2}\right). \end{array}$$

Hence, $\cap_{i\in I}\zeta_{i}$ is a fuzzy ideal of A .

Remark 4.9 *The union of fuzzy ideals of* A *is not a fuzzy ideal of* A .

Example 4.10 *Let* $A=\left\{0,\;\gamma_{1},\;\gamma_{2},\;\gamma_{3}\right\}$ *with* $0\leq \gamma_{1},\;\gamma_{2}\leq \gamma_{3}$ *and elements* $\gamma_{1},\;\gamma_{2}$ *are incomparable. Define* ⊗ *and* + *by the following tables.*

**Table:** 

⊗	0	$\gamma_{1}$	$\gamma_{2}$	$\gamma_{3}$
0	0	$\gamma_{1}$	$\gamma_{2}$	$\gamma_{3}$
$\gamma_{1}$	$\gamma_{1}$	0	$\gamma_{3}$	$\gamma_{2}$
$\gamma_{2}$	$\gamma_{2}$	$\gamma_{3}$	0	$\gamma_{1}$
$\gamma_{3}$	$\gamma_{3}$	$\gamma_{2}$	$\gamma_{1}$	0

**Table:** 

+	0	$\gamma_{1}$	$\gamma_{2}$	$\gamma_{3}$
0	0	$\gamma_{1}$	$\gamma_{2}$	$\gamma_{3}$
$\gamma_{1}$	$\gamma_{1}$	$\gamma_{1}$	$\gamma_{3}$	$\gamma_{3}$
$\gamma_{2}$	$\gamma_{2}$	$\gamma_{3}$	$\gamma_{2}$	$\gamma_{3}$
$\gamma_{3}$	$\gamma_{3}$	$\gamma_{3}$	$\gamma_{3}$	$\gamma_{3}$

*Then,* A *is an autometrized algebra. Define a fuzzy subset* ζ *and* α *of* A *by:*

**Table:** 

	0	$\gamma_{1}$	$\gamma_{2}$	$\gamma_{3}$
ζ	0.5	0.3	0.5	0.3
α	0.5	0.5	0.4	0.4
ζ∪α	0.5	0.5	0.5	0.4

*Then* ζ *and* α *are fuzzy ideals of* A *. But the union* ζ∪α *is not a fuzzy ideals of* A *. Since* $\zeta \cup \alpha \left(\gamma_{1}+\gamma_{2}\right)=\zeta \cup \alpha \left(\gamma_{3}\right)=0.4<0.5=\min\left\{\zeta \cup \alpha \left(\gamma_{1}\right),\zeta \cup \alpha \left(\gamma_{2}\right)\right\}$ .

Remark 4.11 *The union of any chain set of fuzzy ideals of* A *is also fuzzy ideal of* A .

Proof.
Let $\left\{\zeta_{i}|i\in I\right\}$ be a family of fuzzy ideals of autometrized algebra A . Since it is a chain $\cup_{i\in I}\zeta_{i}=\zeta_{k}$ , for some k∈I . Then $\cup_{i\in I}\zeta_{i}$ is a fuzzy ideal of A .

Theorem 4.12 *A homomorphic pre-image of a fuzzy ideal of* A *is also a fuzzy ideal of* A .

Proof.
Let f:A→B be a homomorphism. Let α a fuzzy ideal of B and ζ the pre-image of α under f , then α(f(γ))=ζ(γ) for all γ∈A . To show that ζ is a fuzzy ideal of A .

(i) Let $\gamma_{1},\gamma_{2}\in A$ .

$$\begin{array}{l} \zeta \left(\gamma_{1}+\gamma_{2}\right)=\alpha \left(f\left(\gamma_{1}+\gamma_{2}\right)\right).\\ \;=\alpha \left(f\left(\gamma_{1}\right)+f\left(\gamma_{2}\right)\right).\\ \;\geq \min\left\{\alpha \left(f\left(\gamma_{1}\right)\right),\alpha \left(f\left(\gamma_{2}\right)\right)\right\}.\\ \;=\min\left\{\zeta \left(\gamma_{1}\right),\zeta \left(\gamma_{2}\right)\right\}. \end{array}$$

(ii) Let $\gamma_{1},\gamma_{2}\in A$ and $\gamma_{1}⊗0\leq \gamma_{2}⊗0$ . Since f is homomorphism; $f\left(\gamma_{1}⊗0\right)\leq f\left(\gamma_{2}⊗0\right)$ . So, $f\left(\gamma_{1}\right)⊗0\leq f\left(\gamma_{2}\right)⊗0$ . Since $f\left(\gamma_{1}\right),\;f\left(\gamma_{2}\right)\in B$ ; $\alpha \left(f\left(\gamma_{1}\right)\right)\geq \alpha \left(f\left(\gamma_{2}\right)\right)$ . Therefore, $\zeta \left(\gamma_{1}\right)\geq \zeta \left(\gamma_{2}\right)$ . Hence, ζ is a fuzzy ideal of A .

Theorem 4.13 *Let* f:A→B *be an epimorphism and order reversing. For every fuzzy ideal* ζ *of* A *with sup property,* f(ζ) *is a fuzzy ideal of* B .

Proof.
To show that f(ζ) is a fuzzy ideal of B . By definition $\alpha \left(\gamma_{2}^{\prime }\right)=f\left(\zeta \right)\left(\gamma_{2}^{\prime }\right)=\sup\left\{\zeta \left(\gamma_{1}\right)|\gamma_{1}\in f^{-1}\left(\gamma_{2}^{\prime }\right)\right\}$ , for all $\gamma_{2}^{\prime }\in B$ ( sup∅=0 ).

(i) Let $\gamma^{\prime },\eta^{\prime }\in B$ . Then, there exist $\gamma_{0},\eta_{0}\in A$ such that $f\left(\gamma_{0}\right)=\gamma^{\prime }$ and $f\left(\eta_{0}\right)=\eta^{\prime }$ . So, $\gamma_{0}\in f^{-1}\left(\gamma^{\prime }\right)$ and $\eta_{0}\in f^{-1}\left(\eta^{\prime }\right)$ . Now, consider

$$\begin{array}{l} \zeta \left(\gamma_{0}+\eta_{0}\right)=\alpha \left(f\left(\gamma_{0}+\eta_{0}\right)\right)=\alpha \left(f\left(\gamma_{0}\right)+f\left(\eta_{0}\right)\right)=\alpha \left(\gamma^{\prime }+\eta^{\prime }\right)=\underset{\gamma_{0}+\eta_{0}\in f^{-1}\left(\gamma^{\prime }+\eta^{\prime }\right)}{\sup}\zeta \left(\gamma_{0}+\eta_{0}\right).\\ \zeta \left(\gamma_{0}\right)=\alpha \left(f\left(\gamma_{0}\right)\right)=\alpha \left(\gamma^{\prime }\right)=\underset{\gamma_{0}\in f^{-1}\left(\gamma^{\prime }\right)}{\sup}\zeta \left(\gamma_{0}\right). \end{array}$$

Also,

$$\begin{array}{l} \alpha \left(\gamma^{\prime }+\eta^{\prime }\right)=\underset{\pi \in f^{-1}\left(\gamma^{\prime }+\eta^{\prime }\right)}{\sup}\zeta \left(\pi \right).\\ \;\geq \min\left\{\zeta \left(\gamma_{0}\right),\zeta \left(\eta_{0}\right)\right\}.\\ \;=\min\left\{\underset{\pi \in f^{-1}\left(\gamma^{\prime }\right)}{\sup}\zeta \left(\gamma_{0}\right),\underset{\pi \in f^{-1}\left(\eta^{\prime }\right)}{\sup}\zeta \left(\eta_{0}\right)\right\}.\\ \;=\min\left\{\alpha \left(\gamma^{\prime }\right),\alpha \left(\eta^{\prime }\right)\right\}. \end{array}$$

(ii) Let $\gamma^{\prime },\eta^{\prime }\in ℬ$ and $\gamma^{\prime }⊗0\leq \eta^{\prime }⊗0$ . To show that $\alpha \left(\gamma^{\prime }\right)\geq \alpha \left(\eta^{\prime }\right)$ . Then, there exist $\gamma_{0},\eta_{0}\in A$ such that $f\left(\gamma_{0}\right)=\gamma^{\prime }$ and $f\left(\eta_{0}\right)=\eta^{\prime }$ . So, $\gamma_{0}\in f^{-1}\left(\gamma^{\prime }\right)$ and $\eta_{0}\in f^{-1}\left(\eta^{\prime }\right)$ .
  Since f is order reversing;

$$\begin{array}{l} f\left(\gamma_{0}\right)⊗0\leq f\left(\eta_{0}\right)⊗0.\\ \Rightarrow f\left(\gamma_{0}⊗0\right)\leq f\left(\eta_{0}⊗0\right).\\ \Rightarrow \gamma_{0}⊗0\leq \eta_{0}⊗0.\\ \Rightarrow \zeta \left(\gamma_{0}\right)\geq \zeta \left(\eta_{0}\right).\\ \Rightarrow \alpha \left(f\left(\gamma_{0}\right)\right)\geq \alpha \left(f\left(\eta_{0}\right)\right).\\ \Rightarrow \alpha \left(\gamma^{\prime }\right)\geq \alpha \left(\eta^{\prime }\right). \end{array}$$

Hence, α is a fuzzy ideal of B .

## 5. Fuzzy congruence of autometrized algebra

This section provides an introduction to fuzzy congruence relations.

Let A be an autometrized algebra. A fuzzy relation on A is a mapping Φ:A×A→[0,1] .

Definition 5.1 *If* Φ *is a fuzzy equivalent relation on* A *, then*

(a)
$\Phi \left(\gamma_{1},\;\gamma_{1}\right)=\sup\left\{\Phi \left(\gamma_{2},\;\gamma_{3}\right)|\gamma_{2},\;\gamma_{3}\in A\right\}$ *.(reflexive)*
(b)
$\Phi \left(\gamma_{1},\;\gamma_{2}\right)=\Phi \left(\gamma_{2},\;\gamma_{1}\right)$ *.(symmetric)*
(c)
$\Phi \left(\gamma_{1},\;\gamma_{3}\right)\geq \min\left\{\Phi \left(\gamma_{1},\;\gamma_{2}\right),\;\Phi \left(\gamma_{2},\;\gamma_{3}\right)\right\}$ *.(transitive)*

Definition 5.2 *A fuzzy equivalence relation* Φ *on* A *is called a fuzzy congruence relation on* A *if*

(a)
$\Phi \left(\gamma_{1}+\gamma_{3},\;\gamma_{2}+\gamma_{4}\right)\geq \min\left\{\Phi \left(\gamma_{1},\;\gamma_{2}\right),\;\Phi \left(\gamma_{3},\;\gamma_{4}\right)\right\}\;\forall \gamma_{1},\;\gamma_{2},\;\gamma_{3},\;\gamma_{4}\in A$ *,*
(b)
$\Phi \left(\gamma_{1}⊗\gamma_{3},\;\gamma_{2}⊗\gamma_{4}\right)\geq \min\left\{\Phi \left(\gamma_{1},\;\gamma_{2}\right),\;\Phi \left(\gamma_{3},\;\gamma_{4}\right)\right\}\;\forall \gamma_{1},\;\gamma_{2},\;\gamma_{3},\;\gamma_{4}\in A$ *,*
(c) *For any* $\gamma_{1},\;\gamma_{2},\;\gamma_{3},\;\gamma_{4}\in A$ *, if* $\gamma_{3}⊗\gamma_{4}\leq \gamma_{1}⊗\gamma_{2}$ *, then* $\Phi \left(\gamma_{3},\;\gamma_{4}\right)\geq \Phi \left(\gamma_{1},\;\gamma_{2}\right)$ .

Let Φ be a fuzzy relation on A . Consider $\Phi_{\pi }=\left\{\left(\gamma_{1},\;\gamma_{2}\right)\in A\times A|\Phi \left(\gamma_{1},\;\gamma_{2}\right)\geq \pi \right\}$ , π∈[0,1] .

Theorem 5.3 *Let* Φ *be a fuzzy relation on* A . Φ *is a fuzzy congruence relation on* A *if and only if for all* π∈[0,1] *,* $\Phi_{\pi }$ *is either empty or a congruence relation on* A .

Proof.
Assume that Φ is a fuzzy congruence relation on A .

(i) Since $\Phi_{\pi }$ is a nonempty; let $\left(\gamma_{1},\;\gamma_{2}\right)\in \Phi_{\pi }$ . So, $\Phi \left(\gamma_{1},\;\gamma_{2}\right)\geq \pi$ . But $\Phi \left(\gamma_{1},\;\gamma_{1}\right)=\sup\left\{\Phi \left(\gamma_{2},\;\gamma_{3}\right)|\gamma_{2},\;\gamma_{3}\in A\right\}\geq \Phi \left(\gamma_{1},\;\gamma_{2}\right)\geq \pi$ . So, $\Phi \left(\gamma_{1},\;\gamma_{1}\right)\geq \pi$ . Therefore, $\left(\gamma_{1},\;\gamma_{1}\right)\in \Phi_{\pi }$ .
(ii) Let $\left(\gamma_{1},\;\gamma_{2}\right)\in \Phi_{\pi }$ . So, $\Phi \left(\gamma_{1},\;\gamma_{2}\right)\geq \pi$ . Clearly, $\Phi \left(\gamma_{1},\;\gamma_{2}\right)=\Phi \left(\gamma_{2},\;\gamma_{1}\right)\geq \pi$ . So, $\Phi \left(\gamma_{2},\;\gamma_{1}\right)\geq \pi$ . Therefore, $\left(\gamma_{2},\;\gamma_{1}\right)\in \Phi_{\pi }$ .
(iii) Let $\left(\gamma_{1},\;\gamma_{2}\right),\;\left(\gamma_{2},\;\gamma_{3}\right)\in \Phi_{\pi }$ . Then, $\Phi \left(\gamma_{1},\;\gamma_{2}\right)\geq \pi$ and $\Phi \left(\gamma_{2},\;\gamma_{3}\right)\geq \pi$ . Clearly, $\min\left\{\Phi \left(\gamma_{1},\;\gamma_{2}\right),\;\Phi \left(\gamma_{2},\;\gamma_{3}\right)\right\}\geq \pi$ . Since Φ is a fuzzy congruence; $\Phi \left(\gamma_{1},\;\gamma_{3}\right)\geq \min\left\{\Phi \left(\gamma_{1},\;\gamma_{2}\right),\;\Phi \left(\gamma_{2},\;\gamma_{3}\right)\right\}\geq \pi$ . Therefore, $\left(\gamma_{1},\;\gamma_{3}\right)\in \Phi_{\pi }$ .
Therefore, $\Phi_{\pi }$ is an equivalence relation.
  (a) Let $\left(\gamma_{1},\;\gamma_{2}\right),\;\left(\gamma_{3},\;\gamma_{4}\right)\in \Phi_{\pi }$ . So, $\Phi \left(\gamma_{1},\;\gamma_{2}\right)\geq \pi$ and $\Phi \left(\gamma_{3},\;\gamma_{4}\right)\geq \pi$ . Since Φ is a fuzzy congruence relation on A ; $\Phi \left(\gamma_{1}+\gamma_{3},\;\gamma_{2}+\gamma_{4}\right)\geq \min\left\{\Phi \left(\gamma_{1},\;\gamma_{2}\right),\;\Phi \left(\gamma_{3},\;\gamma_{4}\right)\right\}\geq \pi$ . Therefore, $\left(\gamma_{1}+\gamma_{3},\;\gamma_{2}+\gamma_{4}\right)\in \Phi_{\pi }$ .
  (b) Let $\left(\gamma_{1},\;\gamma_{2}\right),\;\left(\gamma_{3},\;\gamma_{4}\right)\in \Phi_{\pi }$ . So, $\Phi \left(\gamma_{1},\;\gamma_{2}\right)\geq \pi$ and $\Phi \left(\gamma_{3},\;\gamma_{4}\right)\geq \pi$ . Since Φ is a fuzzy congruence relation on A ; $\Phi \left(\gamma_{1}⊗\gamma_{3},\;\gamma_{2}⊗\gamma_{4}\right)\geq \min\left\{\Phi \left(\gamma_{1},\;\gamma_{2}\right),\;\Phi \left(\gamma_{3},\;\gamma_{4}\right)\right\}\geq \pi$ . Therefore, $\left(\gamma_{1}⊗\gamma_{3},\;\gamma_{2}⊗\gamma_{4}\right)\in \Phi_{\pi }$ .
  (c) Let $\left(\gamma_{1},\;\gamma_{2}\right)\in \Phi_{\pi }$ and $\gamma_{3}⊗\gamma_{4}\leq \gamma_{1}⊗\gamma_{2}$ . Clearly, $\left(\gamma_{1},\;\gamma_{2}\right)\geq \pi$ . Since Φ is a fuzzy congruence relation on A ; then $\Phi \left(\gamma_{3},\;\gamma_{4}\right)\geq \Phi \left(\gamma_{1},\;\gamma_{2}\right)\geq \pi$ . Therefore, $\left(\gamma_{3},\;\gamma_{4}\right)\in \Phi_{\pi }$ . Hence, $\Phi_{\pi }$ is a congruence relation on A .

Conversely, $\Phi_{\pi }$ is a congruence relation on A . To show that Φ is a fuzzy congruence relation on A .

(i) We know that for any $\gamma_{1},\;\gamma_{2},\;\gamma_{3}\in A$ , $\gamma_{3}⊗\gamma_{3}=0\leq \gamma_{1}⊗\gamma_{2}$ . Take $\pi =\Phi \left(\gamma_{1},\;\gamma_{2}\right)$ . So, $\left(\gamma_{1},\;\gamma_{2}\right)\in \Phi_{\pi }$ . Since $\Phi_{\pi }$ is a congruence relation; $\left(\gamma_{3},\;\gamma_{3}\right)\in \Phi_{\pi }$ . Then, $\Phi \left(\gamma_{3},\;\gamma_{3}\right)\geq \pi =\Phi \left(\gamma_{1},\;\gamma_{2}\right)$ . Therefore, $\Phi \left(\gamma_{3},\;\gamma_{3}\right)=\sup\left\{\Phi \left(\gamma_{1},\;\gamma_{2}\right)|\gamma_{1},\;\gamma_{2}\in A\right\}$ .
(ii) Let $\gamma_{1},\;\gamma_{2}\in A$ . Take $\pi =\Phi \left(\gamma_{1},\;\gamma_{2}\right)$ . So, $\left(\gamma_{1},\;\gamma_{2}\right)\in \Phi_{\pi }$ . Since $\Phi_{\pi }$ is a congruence; $\left(\gamma_{2},\;\gamma_{1}\right)\in \Phi_{\pi }$ . So, $\Phi \left(\gamma_{2},\;\gamma_{1}\right)\geq \pi$ . Clearly, $\Phi \left(\gamma_{2},\;\gamma_{1}\right)\geq \Phi \left(\gamma_{1},\;\gamma_{2}\right)$ . Again, take $\pi =\Phi \left(\gamma_{2},\;\gamma_{1}\right)$ . So, $\left(\gamma_{2},\;\gamma_{1}\right)\in \Phi_{\pi }$ . Since $\Phi_{\pi }$ is a congruence; $\left(\gamma_{1},\;\gamma_{2}\right)\in \Phi_{\pi }$ . So, $\Phi \left(\gamma_{1},\;\gamma_{2}\right)\geq \pi$ . Clearly, $\Phi \left(\gamma_{1},\;\gamma_{2}\right)\geq \Phi \left(\gamma_{2},\;\gamma_{1}\right)$ . Consequently, $\Phi \left(\gamma_{1},\;\gamma_{2}\right)=\Phi \left(\gamma_{2},\;\gamma_{1}\right)$ .
(iii) Let $\gamma_{1},\;\gamma_{2},\;\gamma_{3}\in A$ . Take $\pi =\min\left\{\Phi \left(\gamma_{1},\;\gamma_{2}\right),\;\Phi \left(\gamma_{2},\;\gamma_{3}\right)\right\}$ . This implies that $\left(\gamma_{1},\;\gamma_{2}\right),\;\left(\gamma_{2},\;\gamma_{3}\right)\in \Phi_{\pi }$ . Since $\Phi_{\pi }$ is a congruence relation; $\left(\gamma_{1},\;\gamma_{3}\right)\in \Phi_{\pi }$ . Therefore, $\Phi \left(\gamma_{1},\;\gamma_{3}\right)\geq \pi$ . Thus, $\Phi \left(\gamma_{1},\;\gamma_{3}\right)\geq \min\left\{\Phi \left(\gamma_{1},\;\gamma_{2}\right),\;\Phi \left(\gamma_{2},\;\gamma_{3}\right)\right\}$ .
Therefore, Φ is an equivalence relation.
  (a) Let $\gamma_{1},\;\gamma_{2},\;\gamma_{3},\;\gamma_{4}\in A$ . Take $\pi =\min\left\{\Phi \left(\gamma_{1},\;\gamma_{2}\right),\;\Phi \left(\gamma_{3},\;\gamma_{4}\right)\right\}$ . Therefore, $\left(\gamma_{1},\;\gamma_{2}\right)\in \Phi_{\pi }$ and $\left(\gamma_{3},\;\gamma_{4}\right)\in \Phi_{\pi }$ . Since $\Phi_{\pi }$ is a congruence relation on A ; $\left(\gamma_{1}+\gamma_{3},\;\gamma_{2}+\gamma_{4}\right)\in \Phi_{\pi }$ . As a result, $\Phi \left(\gamma_{1}+\gamma_{3},\;\gamma_{2}+\gamma_{4}\right)\geq \pi =\min\left\{\Phi \left(\gamma_{1},\;\gamma_{2}\right),\;\Phi \left(\gamma_{3},\;\gamma_{4}\right)\right\}$ .
  (b) Let $\gamma_{1},\;\gamma_{2},\;\gamma_{3},\;\gamma_{4}\in A$ . Take $\pi =\min\left\{\Phi \left(\gamma_{1},\;\gamma_{2}\right),\;\Phi \left(\gamma_{3},\;\gamma_{4}\right)\right\}$ . Therefore, $\left(\gamma_{1},\;\gamma_{2}\right)\in \Phi_{\pi }$ and $\left(\gamma_{3},\;\gamma_{4}\right)\in \Phi_{\pi }$ . Since $\Phi_{\pi }$ is a congruence relation on A ; $\left(\gamma_{1}⊗\gamma_{3},\;\gamma_{2}⊗\gamma_{4}\right)\in \Phi_{\pi }$ . As a result, $\Phi \left(\gamma_{1}⊗\gamma_{3},\;\gamma_{2}⊗\gamma_{4}\right)\geq \pi =\min\left\{\Phi \left(\gamma_{1},\;\gamma_{2}\right),\;\Phi \left(\gamma_{3},\;\gamma_{4}\right)\right\}$ .
  (c) Let $\gamma_{1},\;\gamma_{2},\;\gamma_{3},\;\gamma_{4}\in A$ and suppose that $\gamma_{3}⊗\gamma_{4}\leq \gamma_{1}⊗\gamma_{2}$ . Take $\pi =\Phi \left(\gamma_{1},\;\gamma_{2}\right)$ . Therefore, $\left(\gamma_{1},\;\gamma_{2}\right)\in \Phi_{\pi }$ . Since $\Phi_{\pi }$ is a congruence relation on A ; $\left(\gamma_{3},\;\gamma_{4}\right)\in \Phi_{\pi }$ . Therefore, $\Phi \left(\gamma_{3},\;\gamma_{4}\right)\geq \pi =\Phi \left(\gamma_{1},\;\gamma_{2}\right)$ . Hence, Φ is a fuzzy congruence relation on A .
  For a fuzzy congruence relation Φ , the fuzzy subset $\Phi^{\gamma_{1}}:A\to \left[0,\;1\right]$ , which is defined by $\Phi^{\gamma_{1}}\left(\gamma_{2}\right)=\Phi \left(\gamma_{1},\;\gamma_{2}\right)$ , is called the fuzzy congruence class containing $\gamma_{1}$ .

Theorem 5.4 *For any fuzzy congruence relation* Φ *on* A *,* $\Phi^{0}=\zeta_{\Phi }$ *is a fuzzy ideal of* A .

Proof.
Let x∈A . Then,

(i) It is clear that, $\Phi^{0}\left(0\right)=\Phi \left(0,\;0\right)$ . We know that $0⊗0\leq \gamma_{1}⊗0$ for all $\gamma_{1}\in A$ . Since Φ is a fuzzy congruence relation; $\Phi \left(0,\;0\right)\geq \Phi \left(0,\;\gamma_{1}\right)$ . This implies that, $\Phi^{0}\left(0\right)\geq \Phi^{0}\left(\gamma_{1}\right)$ .
(ii)
$\Phi^{0}\left(\gamma_{1}+\gamma_{2}\right)=\Phi \left(0,\;\gamma_{1}+\gamma_{2}\right)=\Phi \left(0+0,\;\gamma_{1}+\gamma_{2}\right)$ . Since Φ is a fuzzy congruence relation; $\Phi^{0}\left(\gamma_{1}+\gamma_{2}\right)=\Phi \left(0+0,\;\gamma_{1}+\gamma_{2}\right)\geq \min\left\{\Phi \left(0,\;\gamma_{1}\right),\Phi \left(0,\;\gamma_{2}\right)\right\}=\min\left\{\Phi^{0}\left(\gamma_{1}\right),\Phi^{0}\left(\gamma_{2}\right)\right\}$ .
(iii) Let $\gamma_{1},\;\gamma_{2}\in A$ . Suppose $\gamma_{1}⊗0\leq \gamma_{2}⊗0$ . Since Φ is a fuzzy congruence relation; $\Phi \left(\gamma_{1},\;0\right)\geq \Phi \left(\gamma_{2},\;0\right)$ . Therefore, $\Phi^{0}\left(\gamma_{1}\right)\geq \Phi^{0}\left(\gamma_{2}\right)$ . Hence, $\Phi^{0}$ is a fuzzy ideal of A .

Theorem 5.5 *Let* A *is a normal autometrized algebra. Let* ζ *be a fuzzy ideal of* A *and a fuzzy relation* Φ *on* A *defined by* $\Phi_{\zeta }=\Phi \left(\gamma_{1},\;\gamma_{2}\right)=\zeta \left(\gamma_{1}⊗\gamma_{2}\right)$ *. Then,* $\Phi_{\zeta }$ *is a fuzzy congruence relation on* A .

Proof.
Let ζ be a fuzzy ideal of A . Therefore,

(i) It is clear that, $\zeta \left(0\right)\geq \zeta \left(\gamma_{1}\right)$ for all $\gamma_{1}\in A$ . So, $\Phi \left(\gamma_{1},\;\gamma_{1}\right)=\zeta \left(\gamma_{1}⊗\gamma_{1}\right)=\zeta \left(0\right)\geq \zeta \left(\gamma_{1}\right)$ for all $\gamma_{1}\in A$ . Therefore, $\Phi \left(\gamma_{1},\;\gamma_{1}\right)\geq \zeta \left(\gamma_{2}⊗\gamma_{3}\right)=\Phi \left(\gamma_{2},\;\gamma_{3}\right)$ . Hence, $\Phi \left(\gamma_{1},\;\gamma_{1}\right)=\sup\left\{\Phi \left(\gamma_{2},\;\gamma_{3}\right)|\gamma_{2},\;\gamma_{3}\in A\right\}$ .
(ii) It is clear that; $\Phi \left(\gamma_{1},\;\gamma_{2}\right)=\zeta \left(\gamma_{1}⊗\gamma_{2}\right)=\zeta \left(\gamma_{2}⊗\gamma_{1}\right)=\Phi \left(\gamma_{2},\;\gamma_{1}\right)$ .
(iii) By metric property; $\gamma_{1}⊗\gamma_{2}\leq \gamma_{1}⊗\gamma_{3}+\gamma_{3}⊗\gamma_{2}$ . And also, A is a normal autometrized algebra; implies that $\left(\gamma_{1}⊗\gamma_{2}\right)⊗0\leq \left(\gamma_{1}⊗\gamma_{3}+\gamma_{3}⊗\gamma_{2}\right)⊗0$ . Since ζ is a fuzzy ideal; $\zeta \left(\gamma_{1}⊗\gamma_{2}\right)\geq \zeta \left(\gamma_{1}⊗\gamma_{3}+\gamma_{3}⊗\gamma_{2}\right)$ . Again, ζ is a fuzzy ideal; $\zeta \left(\gamma_{1}⊗\gamma_{2}\right)\geq \min\left\{\zeta \left(\gamma_{1}⊗\gamma_{3}\right),\;\zeta \left(\gamma_{2}⊗\gamma_{3}\right)\right\}$ . Thus, $\Phi \left(\gamma_{1},\;\gamma_{2}\right)\geq \min\left\{\Phi \left(\gamma_{1},\;\gamma_{3}\right),\;\Phi \left(\gamma_{2},\;\gamma_{3}\right)\right\}$ .
Therefore, $\Phi_{\zeta }$ is an equivalence relation.
  (a) By property of normality; $\left[\left(\gamma_{1}+\gamma_{3}\right)⊗\left(\gamma_{2}+\gamma_{4}\right)\right]⊗0\leq \left[\left(\gamma_{1}⊗\gamma_{2}\right)+\left(\gamma_{3}⊗\gamma_{4}\right)\right]⊗0$ . Since ζ is a fuzzy ideal;

$$\begin{array}{l} \zeta \left(\left(\gamma_{1}+\gamma_{3}\right)⊗\left(\gamma_{2}+\gamma_{4}\right)\right)\geq \zeta \left(\left(\gamma_{1}⊗\gamma_{2}\right)+\left(\gamma_{3}⊗\gamma_{4}\right)\right).\\ \;\Phi \left(\gamma_{1}+\gamma_{3},\;\gamma_{2}+\gamma_{4}\right)\geq \min\left\{\zeta \left(\gamma_{1}⊗\gamma_{2}\right),\;\zeta \left(\gamma_{3}⊗\gamma_{4}\right)\right\}.\\ \;\geq \min\left\{\Phi \left(\gamma_{1},\;\gamma_{2}\right),\;\Phi \left(\gamma_{3},\;\gamma_{4}\right)\right\}. \end{array}$$

(b) By property of normality; $\left[\left(\gamma_{1}⊗\gamma_{3}\right)⊗\left(\gamma_{2}⊗\gamma_{4}\right)\right]⊗0\leq \left[\left(\gamma_{1}⊗\gamma_{2}\right)+\left(\gamma_{3}⊗\gamma_{4}\right)\right]⊗0$ . Since ζ is a fuzzy ideal;

$$\begin{array}{l} \zeta \left(\left(\gamma_{1}⊗\gamma_{3}\right)⊗\left(\gamma_{2}⊗\gamma_{4}\right)\right)\geq \zeta \left(\left(\gamma_{1}⊗\gamma_{2}\right)+\left(\gamma_{3}⊗\gamma_{4}\right)\right).\\ \;\Phi \left(\gamma_{1}⊗\gamma_{3},\;\gamma_{2}⊗\gamma_{4}\right)\geq \min\left\{\zeta \left(\gamma_{1}⊗\gamma_{2}\right),\;\zeta \left(\gamma_{3}⊗\gamma_{4}\right)\right\}.\\ \;\geq \min\left\{\Phi \left(\gamma_{1},\;\gamma_{2}\right),\;\Phi \left(\gamma_{3},\;\gamma_{4}\right)\right\}. \end{array}$$

(c) Let $\gamma_{1},\;\gamma_{2},\;\gamma_{3},\;\gamma_{4}\in A$ and suppose that $\gamma_{3}⊗\gamma_{4}\leq \gamma_{1}⊗\gamma_{2}$ . Since A is a normal autometrized algebra; $\left(\gamma_{3}⊗\gamma_{4}\right)⊗0\leq \left(\gamma_{1}⊗\gamma_{2}\right)⊗0$ . Since ζ is a fuzzy ideal; $\zeta \left(\gamma_{3}⊗\gamma_{4}\right)\geq \zeta \left(\gamma_{1}⊗\gamma_{2}\right)$ . Therefore, $\Phi \left(\gamma_{3},\;\gamma_{4}\right)\geq \Phi \left(\gamma_{1},\;\gamma_{2}\right)$ . Hence, $\Phi_{\zeta }$ is a fuzzy congruence relation on A .

Theorem 5.6 *Let* A *is a normal autometrized algebra. Then,* $\Phi_{\zeta }^{\gamma_{1}}=\Phi_{\zeta }^{\gamma_{2}}$ *if and only if* $\zeta \left(\gamma_{1}⊗\gamma_{2}\right)=\zeta \left(0\right)$ .

Proof.
Let $\Phi_{\zeta }^{\gamma_{1}}=\Phi_{\zeta }^{\gamma_{2}}$ for any $\gamma_{1},\;\gamma_{2}\in A$ . Clearly, $\Phi_{\zeta }^{\gamma_{1}}\left(\gamma_{1}\right)=\Phi_{\zeta }^{\gamma_{2}}\left(\gamma_{1}\right)$ . Therefore, $\Phi_{\zeta }^{\gamma_{1}}\left(\gamma_{1}\right)=\Phi \left(\gamma_{1},\;\gamma_{1}\right)=\zeta \left(\gamma_{1}⊗\gamma_{1}\right)=\zeta \left(0\right)$ and $\Phi_{\zeta }^{\gamma_{1}}\left(\gamma_{2}\right)=\Phi \left(\gamma_{1},\;\gamma_{2}\right)=\zeta \left(\gamma_{1}⊗\gamma_{2}\right)$ . This implies that; $\zeta \left(0\right)=\zeta \left(\gamma_{1}⊗\gamma_{2}\right)$ .

Conversely, for any $\gamma_{3}\in A$ ; $\Phi_{\zeta }^{\gamma_{1}}\left(\gamma_{3}\right)=\Phi \left(\gamma_{1},\;\gamma_{3}\right)=\zeta \left(\gamma_{1}⊗\gamma_{3}\right)$ . By using the property of metric; $\gamma_{1}⊗\gamma_{3}\leq \gamma_{1}⊗\gamma_{2}+\gamma_{2}⊗\gamma_{3}$ . And also, A is a normal autometrized algebra; implies that $\left(\gamma_{1}⊗\gamma_{3}\right)⊗0\leq \left(\gamma_{1}⊗\gamma_{2}+\gamma_{2}⊗\gamma_{3}\right)⊗0$ . Since ζ is fuzzy ideal;

$$\begin{array}{l} \zeta \left(\gamma_{1}⊗\gamma_{3}\right)\geq \zeta \left(\gamma_{1}⊗\gamma_{2}+\gamma_{3}⊗\gamma_{2}\right).\\ \;\geq \min\left\{\zeta \left(\gamma_{1}⊗\gamma_{2}\right),\;\zeta \left(\gamma_{3}⊗\gamma_{2}\right)\right\}.\\ \;\geq \min\left\{\zeta \left(0\right),\;\zeta \left(\gamma_{3}⊗\gamma_{2}\right)\right\}.\left[\text{Since}\;\zeta \left(\gamma_{1}⊗\gamma_{2}\right)=\zeta \left(0\right)\right].\\ \zeta \left(\gamma_{1}⊗\gamma_{3}\right)\geq \zeta \left(\gamma_{3}⊗\gamma_{2}\right). \end{array}$$ (1)

Similarly, by using the property of metric; $\gamma_{2}⊗\gamma_{3}\leq \gamma_{1}⊗\gamma_{2}+\gamma_{1}⊗\gamma_{3}$ . And also, A is a normal autometrized algebra; implies that $\left(\gamma_{2}⊗\gamma_{3}\right)⊗0\leq \left(\gamma_{1}⊗\gamma_{2}+\gamma_{1}⊗\gamma_{3}\right)⊗0$ . Since ζ is fuzzy ideal;

$$\begin{array}{l} \zeta \left(\gamma_{2}⊗\gamma_{3}\right)\geq \zeta \left(\gamma_{1}⊗\gamma_{2}+\gamma_{3}⊗\gamma_{1}\right).\\ \;\geq \min\left\{\zeta \left(\gamma_{1}⊗\gamma_{2}\right),\;\zeta \left(\gamma_{3}⊗\gamma_{1}\right)\right\}.\\ \;\geq \min\left\{\zeta \left(0\right),\;\zeta \left(\gamma_{3}⊗\gamma_{1}\right)\right\}.\left[\text{Since}\;\zeta \left(\gamma_{1}⊗\gamma_{2}\right)=\zeta \left(0\right)\right]\\ \zeta \left(\gamma_{2}⊗\gamma_{3}\right)\geq \zeta \left(\gamma_{1}⊗\gamma_{3}\right). \end{array}$$ (2)

By (1) and (2), $\Phi_{\zeta }^{\gamma_{1}}\left(\gamma_{3}\right)=\zeta \left(\gamma_{1}⊗\gamma_{3}\right)=\zeta \left(\gamma_{3}⊗\gamma_{2}\right)=\Phi_{\zeta }^{\gamma_{2}}\left(\gamma_{3}\right)$ . Hence, $\Phi_{\zeta }^{\gamma_{1}}=\Phi_{\zeta }^{\gamma_{2}}$ .

Theorem 5.7 *Let* A *is a normal autometrized algebra. Let* Φ *be a fuzzy congruence and* ζ *be a fuzzy ideal of* A *. Then*

(i)
$\Phi_{\zeta_{\Phi }}=\Phi$ .
(ii)
$\zeta_{\Phi_{\zeta }}=\zeta$ .

Proof.

(i)
$\Phi_{\zeta_{\Phi }}\left(\gamma_{1},\;\gamma_{2}\right)=\zeta_{\Phi }\left(\gamma_{1}⊗\gamma_{2}\right)=\Phi^{0}\left(\gamma_{1}⊗\gamma_{2}\right)=\Phi \left(0,\;\gamma_{1}⊗\gamma_{2}\right)$ for any $\gamma_{1},\;\gamma_{2}\in A$ . Since A is normal; $\left(\gamma_{1}⊗\gamma_{2}\right)⊗0=\gamma_{1}⊗\gamma_{2}$ . This implies that $\Phi \left(0,\;\gamma_{1}⊗\gamma_{2}\right)=\Phi \left(\gamma_{1},\;\gamma_{2}\right)$ . Hence, $\Phi_{\zeta_{\Phi }}=\Phi$ .
(ii)
$\zeta_{\Phi_{\zeta }}\left(\gamma_{1}\right)=\Phi_{\zeta }^{0}\left(\gamma_{1}\right)=\Phi_{\zeta }\left(0,\;\gamma_{1}\right)=\zeta \left(\gamma_{1}⊗0\right)$ for any $\gamma_{1}\in A$ . Since A is normal; $\left(\gamma_{1}⊗0\right)⊗0=\gamma_{1}⊗0$ . This implies that $\zeta \left(\gamma_{1}⊗0\right)=\zeta \left(\gamma_{1}\right)$ . Hence, $\zeta_{\Phi_{\zeta }}=\zeta$ .

Theorem 5.8 *Let* A *be a normal autometrized algebra.*

*Let* FI(A) *= the set of all fuzzy ideals of* A .

*Let* FC(A) *= the set of all fuzzy congruences on* A .

*Then* FI(A) *and* FC(A) *are in one to one correspondence.*

Proof.
Define ψ:FI(A)→FC(A) by $\psi \left(\zeta \right)=\Phi_{\zeta }$ .

Let ζ,α∈FI(A) . Suppose ζ=α . This implies $\Phi_{\zeta }=\Phi_{\alpha }$ . Then ψ(ζ)=ψ(α) . Therefore ψ is well-defined. Now to show that ψ is onto. Let Φ∈FC(A) . Then by theorem (5.4); $\exists \zeta_{\Phi }\in \mathrm{FI}\left(A\right)$ such that:

$$\begin{array}{l} \psi \left(\zeta_{\Phi }\right)=\Phi_{\zeta_{\Phi }}.\\ =\Phi .\left[\text{By}\;\left(i\right)\;\text{of the theorem}\;\left(5.7\right)\right] \end{array}$$

Therefore ψ is onto.

Finally let us show that ψ is one to one. Suppose ψ(ζ)=ψ(α) . This implies that $\Phi_{\zeta }=\Phi_{\alpha }$ . Thus, $\zeta_{\Phi_{\zeta }}=\zeta_{\Phi_{\alpha }}$ . Consider,

$$\zeta_{\Phi_{\zeta }}=\zeta .\left[\text{By}\;\left(\text{ii}\right)\;\text{of the theorem}\;\left(5.7\right)\right]$$

Whence, ζ=α . Hence ψ is one to one and onto. Then ψ is one to one correspondence. Therefore FI(A) and FC(A) are in one to one correspondence.

## 6. Conclusion

This paper presented fuzzy subalgebras of autometrized algebras and examined their properties. We introduced fuzzy ideals of autometrized algebras and provided examples to illustrate our findings. Furthermore, we defined the notions of intersection and union for fuzzy subalgebras and fuzzy ideals in autometrized algebras. We also studied the homomorphisms of both the images and the inverse images of fuzzy subalgebras and ideals. Finally, we introduced fuzzy congruences on autometrized algebras. We showed that in normal autometrized algebra, the set of all fuzzy ideals is in one-to-one correspondence with the set of all fuzzy congruences.

## Author contributions

All authors contributed equally to this manuscript and approved the final version.

## Ethics and consent

Ethics and consent were not necessary.

## Data Availability

This article does not contain any associated data.
