# Supplementary material for: Fuzzy Set Theory Applied on Autometrized Algebra
Source: F1000Res. 2025 Feb 4;14:159. [Version 1] doi: 10.12688/f1000research.161249.1 (PMC11871425; doi:10.12688/f1000research.161249.1)
Supplement: Peer review history

# Reviewer report: Reviewer response for version 1

**DOI:** 10.5256/f1000research.177253.r365974

**Published:** 2025-02-28

**Authors:** Sileshe Gone Korma

**Affiliations:** 1 Hawassa University, Hawassa, Ethiopia

The author introduces the concept of fuzzy subalgebras in autometrized algebras and investigates their characteristics. Additionally, the paper examines fuzzy ideals, providing examples to illustrate key results. It also investigates homomorphisms of fuzzy subalgebras and ideals. Furthermore, the study introduces fuzzy congruences and establishes a one-to-one correspondence between fuzzy ideals and fuzzy congruences in normal autometrized algebras.

 The author should review the points I have highlighted in my comments and address them accordingly to improve clarity and accuracy.

1. Definition 2.2 (line 1) It is better to say; **An autometrized algebra A is called normal if and only if.**
2. Definition 2.5 **seems it is a result not a definition.**
3. Definition 2.9 it is better to say “ **Let A and B are autometrized algebra and f:A** ….”
4. Definition 2.16 (line 3) there must be a space between ***if***  **and** ***f*** ***-1*** ***(*** ***γ*** ***2*** ***)***
5. Before Theorem 3.2, you need to state the equivalent condition for a subset to be a subalgebra, as you use this result in the proof of Theorem 3.2.
6. Theorem 3.2 (on converse part of the proof (i))  it seems typing **error** ***ζ*** ***0*** ***<ζ(*** ***γ*** ***-1*** ***)***  **.** Also, I am **not clear how** ***ζ*** ***0*** ***<*** ***π*** ***-1***  **.**
7. Theorem 3.3 (proof part line 1) make it   .   .   . **to show a fuzzy subalgebra**
8. Remark 3.4 :  it is better to write The union of fuzzy subalgebras of A **may not be** a fuzzy subalgebra of A.
9. Theorem 4.3 (proof converse part (i) line 2) **add { }**
10. Theorem 4.5 (line 1) : Let *I*  be a nonempty subset of A. **Then** ***ζ*** ***I***  **is a fuzzy** ideal of A if and only if I is an ideal of A. Also, for converse part (iii) it seems ***ζ*** ***1*** ***∉I***  **or** ***ζ*** ***2*** ***∉I.***  ****Additionally, the second case does not seem necessary to me.
11. Theorem 4.7 (line 1) It **seems** ***β≤α***
12. **Remark 4.9 : **The union of fuzzy ideals of A **may not be** a fuzzy ideal of A.
13. **Definition 5.**1 : It is better to write “ A fuzzy relation  Φ is called a fuzzy equivalent relation on A”

Is the work clearly and accurately presented and does it cite the current literature?

Yes

If applicable, is the statistical analysis and its interpretation appropriate?

Not applicable

Are all the source data underlying the results available to ensure full reproducibility?

Yes

Is the study design appropriate and is the work technically sound?

Yes

Are the conclusions drawn adequately supported by the results?

Yes

Are sufficient details of methods and analysis provided to allow replication by others?

Yes

Reviewer Expertise:

Algebra, lattice theory, universal algebra, and fuzzy algebra

I confirm that I have read this submission and believe that I have an appropriate level of expertise to confirm that it is of an acceptable scientific standard.

# Reviewer report: Reviewer response for version 1

**DOI:** 10.5256/f1000research.177253.r365973

**Published:** 2025-02-13

**Authors:** Ramazan Yasar

**Affiliations:** 1 Ankara University, Ankara, Turkey

**Summary of the Article :**

 This paper investigates fuzzy subalgebras, fuzzy ideals, and fuzzy congruences within autometrized algebras, presenting rigorous mathematical definitions, theorems, and proofs. The study establishes a bijective correspondence between fuzzy ideals and fuzzy congruences in normal autometrized algebras, contributing significantly to the algebraic theory of fuzzy structures.

**Key Strengths:**

 ✅ Well-structured and logically organized.

 ✅ Clearly defined mathematical framework with precise notation.

 ✅ Theorems and proofs follow established techniques in fuzzy algebra.

 ✅ Relevant literature is well-referenced.

**Areas for Improvement:**

1. **Clarification of Certain Proofs:**
  - The proof of **Theorem 3.3** incorrectly refers to proving an ideal instead of a fuzzy subalgebra. This should be corrected.
  - In **Theorem 5.3**, additional steps should be included to justify why Φπ\Phi_\piΦπ​ is a congruence relation.
  - **Definition 4.1** should better distinguish fuzzy subalgebras from fuzzy ideals to avoid confusion.
2. **Enhancing Readability and Explanation:**
  - The definition of **fuzzy congruence relations** in Section 5 should include a brief discussion of its significance and applications.
  - **Example 3.5** should provide a counterexample demonstrating why the union of fuzzy subalgebras is not necessarily a fuzzy subalgebra.
3. **Conclusion and Impact:**
  - The **conclusion** should explicitly state how the study extends prior research and suggest potential applications in soft computing or other domains.
4. **Reproducibility:**
  - Some examples lack detailed computational verification. Expanding **Example 4.2** with explicit calculations would improve transparency.
  - The study could benefit from providing more concrete computational examples illustrating fuzzy congruences in practice.

**Essential Revisions for Scientific Soundness:**

 ✔ Correct the minor mathematical inconsistencies in proofs and definitions.

 ✔ Provide additional justifications for key theorems.

 ✔ Expand examples with detailed step-by-step calculations.

 ✔ Clarify the real-world significance of fuzzy congruences and their potential applications.

 With these minor revisions, the paper will be more comprehensible and impactful. **I recommend indexing after minor revisions.**

Is the work clearly and accurately presented and does it cite the current literature?

Yes

If applicable, is the statistical analysis and its interpretation appropriate?

Not applicable

Are all the source data underlying the results available to ensure full reproducibility?

Partly

Is the study design appropriate and is the work technically sound?

Yes

Are the conclusions drawn adequately supported by the results?

Partly

Are sufficient details of methods and analysis provided to allow replication by others?

Partly

Reviewer Expertise:

Algebra, Fuzzy Set Theory, Soft Computing, Mathematical Logic, and Theoretical Computer ScienceExpertise in autometrized algebras and fuzzy logic-based mathematical structures, ensuring a thorough review of the algebraic and logical foundations of the paper.Specialized in fuzzy congruences, ideal theory, and homomorphisms within algebraic frameworks.Experience in soft computing applications related to fuzzy algebra and their implications in artificial intelligence and decision-making models.

I confirm that I have read this submission and believe that I have an appropriate level of expertise to confirm that it is of an acceptable scientific standard, however I have significant reservations, as outlined above.

# Reviewer report: Reviewer response for version 1

**DOI:** 10.5256/f1000research.177253.r365978

**Published:** 2025-02-13

**Authors:** Jehad R Kider

**Affiliations:** 1 University of Technology-Iraq, Baghdad, Iraq

“Fuzzy Set Theory Applied on Autometrized Algebra ”

 Thank you for sending me to review this paper.

 Generally, has a good structure and without mathematical mistakes. 

 This paper presented fuzzy subalgebras of autometrized algebras and examined their properties. They introduced fuzzy ideals of autometrized algebras and provided examples to illustrate our findings. Furthermore, they  defined the notions of intersection and union for fuzzy subalgebras and fuzzy ideals in autometrized algebras.  Also they studied the homomorphisms of both the images and the inverse images of fuzzy subalgebras and ideals. Finally, we introduced fuzzy congruences on autometrized algebras

 I suggest for the authors to rewrite the abstract by determine the goals. The manuscript has a few minor typographical errors and grammatical mistakes that should be corrected before acceptance. Taking the above into consideration, I recommend the paper for indexing.

Is the work clearly and accurately presented and does it cite the current literature?

Yes

If applicable, is the statistical analysis and its interpretation appropriate?

Yes

Are all the source data underlying the results available to ensure full reproducibility?

Yes

Is the study design appropriate and is the work technically sound?

Yes

Are the conclusions drawn adequately supported by the results?

Yes

Are sufficient details of methods and analysis provided to allow replication by others?

Yes

Reviewer Expertise:

Fuzzy Metric spaces, Fuzzy Normed Spaces, Fuzzy inner product spaces.

I confirm that I have read this submission and believe that I have an appropriate level of expertise to confirm that it is of an acceptable scientific standard.
